# PHF8 upregulation contributes to autophagic degradation of E-cadherin, epithelial-mesenchymal transition and metastasis in hepatocellular carcinoma

**DOI:** 10.1186/s13046-018-0890-4

**Published:** 2018-09-04

**Authors:** Wuhua Zhou, Li Gong, Qinchuan Wu, Chunyang Xing, Bajin Wei, Tianchi Chen, Yuan Zhou, Shengyong Yin, Bin Jiang, Haiyang Xie, Lin Zhou, Shusen Zheng

**Affiliations:** 1 Division of Hepatobiliary and Pancreatic Surgery, Department of Surgery, The First Affiliated Hospital, School of Medicine, Zhejiang University, Hangzhou, China; 2 NHFPC Key Laboratory of Combined Multi-Organ Transplantation, Hangzhou, China; 3 Key Laboratory of the Diagnosis and Treatment of Organ transplantation, CAMS, Hangzhou, China; 4 Key Laboratory of Organ Transplantation, Hangzhou, Zhejiang Province China; 5 Collaborative Innovation Center for Diagnosis Treatment of Infectious Disease, Zhejiang University, Hangzhou, China; 6 Department of Hepatobiliary and Pancreatic Surgery, Taihe Hospital, Shiyan, China; 7 Department of Endocrinology, Taihe Hospital, Shiyan, China

**Keywords:** Plant homeodomain finger protein 8, PHF8, Epithelial-mesenchymal transition, EMT, Autophagy, Metastasis, Hepatocellular carcinoma, HCC

## Abstract

**Background:**

Plant homeodomain finger protein 8 (PHF8) serves an activator of epithelial-mesenchymal transition (EMT) and is implicated in various tumors. However, little is known about PHF8 roles in hepatocellular carcinoma (HCC) and regulating E-cadherin expression.

**Methods:**

PHF8 expression pattern was investigated by informatic analysis and verified by RT-qPCR and immunochemistry in HCC tissues and cell lines. CCK8, xenograft tumor model, transwell assay, and tandem mCherry-GFP-LC3 fusion protein assay were utilized to assess the effects of PHF8 on proliferation, metastasis and autophagy of HCC cells in vitro and in vivo. ChIP, immunoblot analysis, rescue experiments and inhibitor treatment were used to clarify the mechanism by which PHF8 facilitated EMT, metastasis and autophagy.

**Results:**

PHF8 upregulation was quite prevalent in HCC tissues and closely correlated with worse overall survival and disease-relapse free survival. Furthermore, PHF8-knockdown dramatically suppressed cell growth, migration, invasion and autophagy, and the expression of SNAI1, VIM, N-cadherin and FIP200, and increased E-cadherin level, while PHF8-overexpression led to the opposite results. Additionally, FIP200 augmentation reversed the inhibited effects of PHF8-siliencing on tumor migration, invasion and autophagy. Mechanistically, PHF8 was involved in transcriptionally regulating the expression of SNAI1, VIM and FIP200, rather than N-cadherin and E-cadherin. Noticeably, E-cadherin degradation could be accelerated by PHF8-mediated FIP200-dependent autophagy, a crucial pathway complementary to transcriptional repression of E-cadherin by SNAI1 activation.

**Conclusion:**

These findings suggested that PHF8 played an oncogenic role in facilitating FIP200-dependent autophagic degradation of E-cadherin, EMT and metastasis in HCC. PHF8 might be a promising target for prevention, treatment and prognostic prediction of HCC.

**Electronic supplementary material:**

The online version of this article (10.1186/s13046-018-0890-4) contains supplementary material, which is available to authorized users.

## Background

Hepatocellular carcinoma (HCC), accounting for 90% of all liver cancer cases, is highly prevalent and serves as one of most leading causes of cancer-related deaths worldwide [1]. Although multiple therapeutic strategies and relevant molecular mechanisms have been explored, HCC outcome is still unfavorable mainly due to the high rate of metastasis or relapse [2, 3]. Epithelial-mesenchymal transition (EMT) is believed as an important and complex biological process that regulates tumor invasion and metastasis, characterized by conversion of non-mobile polarized epithelial cells into invasive and metastatic mesenchymal cell, and often modulated by various pathways [4, 5]. Thus, it is necessary to clarify the exact mechanisms of EMT regulation for improving therapeutic strategy for HCC.

Plant homeodomain finger protein 8 (PHF8, also termed KDM7B or JHDM1F) is a member of the histone demethylase and has attracted considerable attention for its wide expression and role as transcriptional co-activator recently [6–11]. PHF8 binds to promoter sites of approximate one third of human genes and activates their expression by erasing repressive histone markers, including H3K9me1/2, H3K27me2 and H4K20me1 [7–9, 12]. These remarkable features imply that the ectopic expression of PHF8 is potentially correlated with genetic and environmental disease such as human cancer. As expected, PHF8 upregulation is a pivotal factor that regulates malignant progression and metastasis in prostate cancer, breast cancer, lung cancer, esophageal squamous cell carcinoma, gastric cancer, leukemia, etc. [11, 13–19]. However, little is known about the expression pattern and roles of PHF8 in HCC.

Previous evidences demonstrated that PHF8 contributes to DNA damage protection, anti-apoptosis, and activation of cell cycle and EMT [13–19]. Notably, great importance has been attached to the implication of PHF8 in transcriptional activating the expression of EMT-related markers, SNAI1 and VIM, and attenuating E-cadherin level [16, 17]. However, it is unclear how PHF8 downregulates E-cadherin expression. E-cadherin inhibition is a fundamental molecular event during EMT due to gene deletion or mutational inactivation, transcriptional repression or autophagic degradation [4, 20, 21]. Autophagy is defined as the evolutionarily conserved and protective “self-eating” process, during which cytoplasmic materials and proteins are engulfed and catalyzed by autophagy-linked lysosome to obtain energy in response to stress [22, 23]. The promotion of autophagy to metastasis of HCC has been substantiated by more recent evidences from in vitro and in vivo studies [24, 25]. Therefore, autophagy cannot be neglected when studying EMT or metastasis in HCC, and it might be helpful to elucidate the exact process of PHF8-induced E-cadherin suppression.

In this study, we reported oncogenic roles of PHF8 in clinical significance and promotion to tumor development, EMT and metastasis in HCC. Our results revealed that the high expression of PHF8 was not only associated with the more aggressive phenotypes and worse outcome, but also enhanced autophagy, invasion and migration through upregulation of FIP200, SNAI1, VIM and CDH2/N-cadherin and E-cadherin attenuation, which could be accelerated by FIP200-dependent autophagy. These findings indicated that PHF8 upregulation may serve as a useful biomarker and a candidate therapeutic target against HCC.

## Methods

### Patient samples

Samples of cancer tissues and matching adjacent normal liver tissues were collected from 226 HCC patients who had received curative hepatectomy from Aug 4th, 2010 to Dec 31st, 2014 at First Affiliated Hospital of Zhejiang University. Written informed content was obtained from each enrolled subject according to the study protocols approved by local ethics committee. Tissue samples were immediately placed in liquid nitrogen after surgical removal and preserved at − 80 °C, and frozen tumor tissues and matching normal tissues from 147 cases were subjected to mRNA extraction for quantitative real-time PCR (qRT-PCR). Samples from 198 cases of above patients with complete clinicopathological and follow-up information were selected for assessing the correlation of PHF8 expression with clinical features and prognosis based on immunohistochemistry analysis. Follow-up was ended on Apr 15th, 2018, with a median of 48 months.

### Cell culture and reagents

Five human liver cancer cell lines (HepG2, MHCC-LM3, Huh7, SMMC-7721, and SK-Hep-1) and two immortalized human normal hepatocytes (L-02 and QYG-7701) maintained at our institute were routinely cultured in Minimum Essential Media (MEM, Gibco, Carlsbad, CA US) supplemented with 10% fetal bovine serum (FBS, Gibco), 100 U/mL penicillin and 100 mg/mL streptomycin (Gibco), and incubated in a humidified atmosphere with 5% CO2 at 37 °C. Earle’s Balanced Salt Solution (EBSS, Gibco), chloroquine (CQ, Selleck, Houston, TX, US) and cycloheximide (CHX, Selleck) were used to establish starvation condition to induce cell autophagy, block autophagy process and inhibit protein synthesis, respectively.

### RNA extraction and qRT-PCR

Total RNA was isolated using TRIzol reagent (Invitrogen, Carlsbad, CA, US), and followed by cDNA synthesis with Taq-Man Reverse Transcription Kit (Takara, Dalian, China) according to manufacturer’s instructions. mRNA expression was detected by SYBR Premix Ex Taq kit (Takara) performed on an ABI Prism 7500 Real-Time System, and relative mRNA amount was normalized against β-actin and calculated by 2^-ΔΔCt^. Sequences of PCR primers were referred from online PrimerBank (https://pga.mgh.harvard.edu/primerbank/) and listed in Additional file 1: Table S1.

### Western-blot

Total protein was extracted from tissues or cells by prechilled RIPA Buffer (Cell Signaling Technology, Danvers, MA, US), and its concentration was measured by BCA Kit (Thermo Scientific™, Rockford, IL, US). An equal amount of protein (40 mg) was resolved via electrophoresis on a precast 4–12% gradient sodium dodecyl sulfate (SDS)- polyacrylamide gel (GenScript, Nanjing, China) and electrotransferred to polyvinylidene fluoride membranes (Thermo Scientific™). Membranes were blocked with 5% non-fat dry milk in Tris-buffered saline with 0.1% Tween 20 (TBST), incubated with primary antibody for overnight, washed by TBST for three times and incubated with secondary antibody of HRP-linked for 1 h (all antibodies for this part were listed in Additional file 2: Table S2). Immunoblot bands were visualized by ECL Kits (Thermo Scientific™) and protein expression was semi-quantitatively analyzed using ImageJ software (National Institutes of Health, Bethesda, MD, US).

### Immunohistochemistry

All specimens were fixed with formalin, embedded by paraffin and cut into 4 μm sections. Tissue slices were subjected to hematoxylin and eosin (H&E) staining or immunohistochemistry (IHC) staining using the two-step method of Dako Envision™ Detection System (DakoCytomation, Glostrup, Denmark). Briefly, tissue slices were incubated with primary antibodies (listed in Additional file 2: Table S2) after deparaffinized, rehydrated and antigen retrieval routinely. All stained slides were reviewed independently by two pathologists. Semi-quantitative analysis of IHC results was described previously [26]. In brief, IHC score was calculated by multiplying staining intensity score (score of 0, 1, 2 and 3 represented negative, weak-positive, moderate-positive and strong-positive, respectively) and the score of relative positive-staining area (score of 0, 1, 2, 3, and 4 indicated positive-staining areas of 0–5%, 6–25%, 26–50%, 51–75% and 76–100%, respectively). IHC score more than 2 was defined as high expression, while the others represented low expression (Additional file 3: Figure S1).

### In vitro proliferation

Cell proliferation was examined using Cell Counting Kit-8 (CCK-8, Dojindo Laboratories, Kumamoto, Japan) according to the manufacturer’s instructions. Cells (5×10^3^ cells per well) were seeded into a 96-well plate and incubated for an indicated time. Absorbance was measured daily for 4 consecutive days at 450 nm.

### Transwell assay

Transwell membrane filter (24-well and 8 μm pore size, Corning) was precoated or uncoated with Matrigel (Becton Dickinson, San Jose, CA, US) for invasion and migration assay, respectively. Then, serum free medium with 5 × 10^4^ HCC cells was added to the upper chamber, and medium containing 20% FBS was placed into the bottom chamber. After incubation for 36-h (migration assay) or 48-h (invasion assay), cells on the underside of membrane were stained with crystal violet (Thermo Scientific™) and migrated or invasive cells were counted in 5 random fields under the microscope.

### RNA inference and transfection of plasmids

HCC cells of 50% confluent were transfected with 0.5ul scramble (shCtrl) or PHF8-specific shRNAs (shPHF8–1# and − 2#) lentiviral particles (GeneCopeia, Guangzhou, China) and maintained in complete medium with 2 mg/ml polybrene (Vigene Bioscience, Jinan, China) for 24 h. Oligo sequences of shRNAs targeting PHF8 were 5-CCGTACAGCTCATTAAAGATC-3 (shPHF8–1#), and 5-GCTTCATGATCGAGTGTGACA-3 (shPHF8–2#) as described previously [16]. These infected cells were treated with 2 μg/ml of puromycin (Invitrogen) to generate stable transfectants. For overexpression assays, empty vector, Flag-PHF8-constructed plasmid (GeneCopeia), or HA-FIP200-constructed plasmid (Vigene Bioscience) were transfected into HCC cells using Lipofectamine® 2000 reagent (Invitrogen) according to the manufacturer’s instructions. According to previous report [27], siRNA of negative control (#1027281, Qiagen, Valencia, CA, US) and targeting- SNAI (#s13185, Thermo Scientific™) were selected for determining the effects of SNAI1 on E-cadherin expression in the context of PHF8 overexpression using Lipofectamine® 2000. Transfection efficiency was determined by qRT-PCR and immunoblot analysis.

### Autophagy induction and ad-mCherry-GFP-LC3 transient transfection

For autophagy analysis, HCC cells with or without PHF8-knockdown, or PHF8-overexpression or combining PHF8-knockdown and FIP200 exogenous overexpression, were infected with adenovirus expressing Ad-mCherry-GFP-LC3 fusion protein (Vigene Bioscience, at multiplicity of infection of 20). After incubation in complete medium for 48 h, cells were treated by starvation medium (EBSS) for 6 h and observed under a fluorescence microscope. Autophagic flux was assessed by manually counting the number of yellow and red dots of each cell in five random fields from the images that merged the red and green channels. Yellow and red dots represented autophagosomes and autolysosomes, respectively.

### ChIP and qRT-PCR

Chromatin immunoprecipitation (ChIP) assay was carried out using SimpleChIP® Enzymatic Chromatin IP Kit (Cell Signaling, Danvers, MA, US) in accordance to the manufacturer’s instructions. Chromatin fragments of HCC cells were immunoprecipitated with 5 μg anti-PHF8 or IgG antibody (Abcam) and then subjected to qRT-PCR using SYBR Premix Ex Taq kit (Takara) performed on an ABI Prism 7500 Real-Time System. Methods for detection and calculation of target amplification were according to previous descriptions [28]: fold enrichment = 2^− (ΔCT^_expt_^- ΔCT^_control_^)^ where ΔCT = CT _anti-PHF8_-_IP_–CT _IgG-IP_, expt = target region, and control = negative control region which was from an intragenic genome. Sequence covering the region of − 2.5 kb upstream and 1.0 kb downstream of TSS was selected for designing primers (Additional file 4: Table S3).

### Tumor cell xenograft model

Animal study was initially approved by Animal Care and Use Committee of Zhejiang University, and conducted under the National Institute Guide for the Care and Use of Laboratory Animals. Five-week old Balb/c male nude mice were purchased from Shanghai Experimental Animal Center of Chinese Academic of Sciences (Shanghai, China). SMMC-7721 cells were used for establishing xenograft tumor model. For tumor growth evaluation, 5 × 10^6^ cells suspended in 50 μl phosphate buffered saline (PBS) were subcutaneously injected to the left flank of each mouse. Tumor size was monitored every 4 days, and mice were sacrificed on 24th day post-graft. Tumor volume was calculated by the formula of (length × width^2^)/ 2. For lung metastasis observation, each mouse was injected with 0.2 ml PBS containing 2 × 10^6^ cells through tail vein, and all lung samples were harvested 6 weeks later and fixed with formalin for histological analysis.

### Statistical analysis

Statistical analysis was carried out using SPSS 22.0 software (Chicago, IL, US), and statistical graphs were generated by GraphPad PRISM 6.01 software (San Diego, CA, US). Independent experiment was repeated three times. Two-tail Student *t* test was used to compare statistical differences between groups. The correlation of PHF8 expression to the clinicopathological parameters and the expression of FIP200 and E-cadherin was analyzed using Pearson Chi-squared test or Fisher’s Exact test. Survival curves were estimated by Kaplan-Meier method and compared by log-rank test. Univariate and multivariate analysis were conducted based on Cox’s proportional regression model to assess independent prognostic factors. *P* values less than 0.05 was defined as statistical significance.

## Results

### PHF8 upregulation is quite prevalent and serves as an independent risk factor for poor prognosis and relapse in HCC

**Fig. 1:**
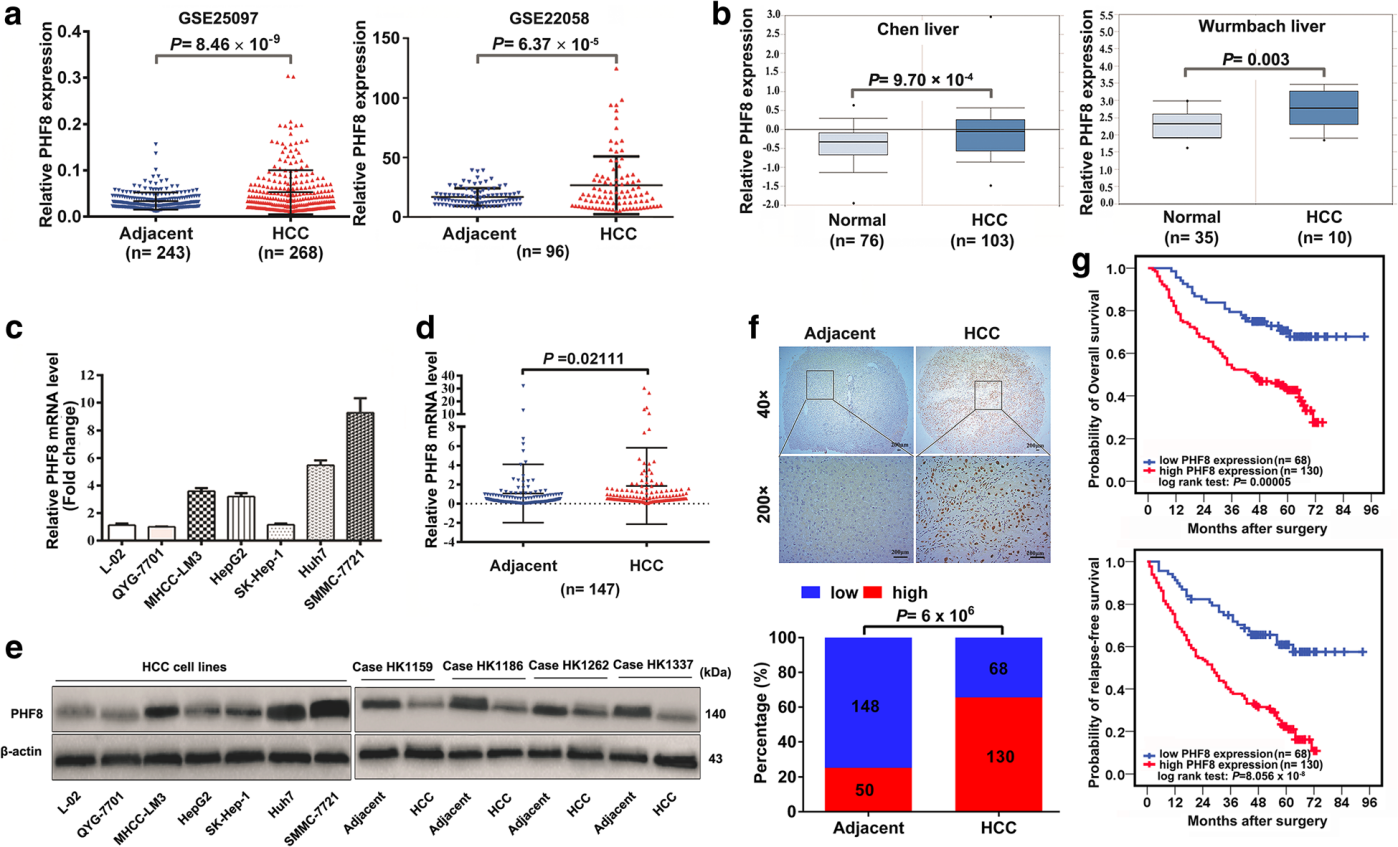
PHF8 expression is prevalently upregulated and indicated a poor prognosis in HCC. **a**, **b** Comparison of PHF8 expression in HCC tissues and normal liver tissues or adjacent normal liver tissues according to the analysis of data from GEO database (GSE25097 and GSE22058), and Oncomine database (Chen liver and Wurmbach liver). **c**, **d** Relative PHF8 mRNA level in HCC cell lines and normal human hepatocytes, and in HCC tissues and adjacent normal liver tissues by qRT-PCR analysis. **e** PHF8 protein expression in HCC cell lines and HCC tissues and adjacent normal tissues by western-blot analysis. β-actin was used as the loading control. **f** Representative immunohistochemical staining for PHF8 (upper panel, magnification, × 40, × 200) and the percentages of low or high PHF8 expression in paired HCC samples (lower panel). **g** Kaplan-Meier analysis of overall survival and relapse-free survival of HCC patients with low (*n* = 68) and high (*n* = 130) expression of PHF8 based on IHC scoring. Data were presented as mean ± SD

To evaluate the expression pattern of PHF8 in HCCs, we initially analyzed two microarray datasets from GEO database (Fig. 1a) and revealed higher expression of PHF8 in HCCs than normal liver tissues. This finding was in line with the analysis of another two datasets from Oncomine Database (Fig. 1b), and supported by the results of remarkable upregulation of PHF8 at both mRNA and protein level in HCC cells compared with normal liver cells, and in HCC tissues in comparison with adjacent normal liver tissues (Fig. 1c-e).

Moreover, the correlation of PHF8 expression with clinicopathological features was investigated in 198 of above HCC patients based on IHC staining. IHC results confirmed that PHF8 expression was increased in HCC tissues (Fig. 1f). High expression of PHF8 was significantly associated with vascular invasion, large tumor size, poor tumor differentiation and advanced tumor stage (Additional file 5: Table S4). Kaplan-Meier analysis demonstrated that high expression of PHF8 conferred a worse overall survival (OS) and relapse-free survival (RFS) in HCC (Fig. 1g). Combining univariate- and multivariate- analysis revealed that PHF8 upregulation, vascular invasion and advanced tumor stage were the independent risk factors for predicting poor OS and RFS (Additional file 6: Table S5).

### PHF8 promotes tumorigenesis and metastasis of HCC cells in vitro and in vivo

**Fig. 2:**
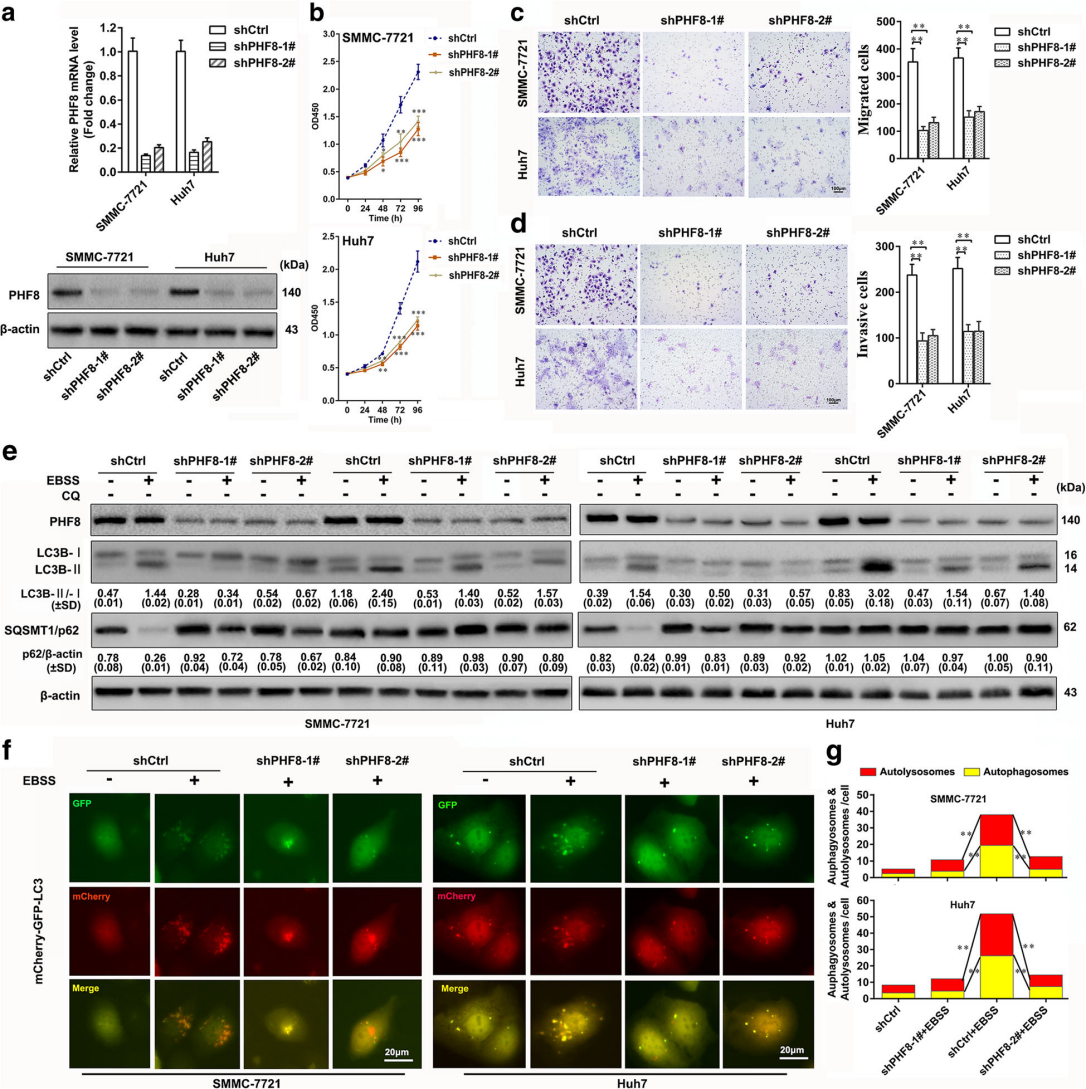
PHF8 knockdown significantly suppresses proliferation, migration, invasion and autophagy of HCC cells in vitro. **a** Determination of transfection efficiency of shRNAs targeting PHF8 in SMMC-7721 and Huh7 cells by qRT-PCR and western-blot assay. Scramble shRNA (shCtrl) was used for negative control. **b** Inhibited proliferation of SMMC-7721 and Huh7 cells in PHF8 knockdown group by CCK8 assasy (*n* = 6). **c**, **d** Representative images and quantification of migrated and invasive cells by transwell assay in SMMC-7721 and Huh7 cells (*n* = 3, magnification, × 100). **e** Representative immunoblot result of autophagy markers, LC3B and p62 in SMMC-7721 and Huh7 cells with PHF8 knockdown. Both cell lines transfected with indicated shRNAs were cultured in complete medium with 10% FBS or EBSS starvation condition with or without CQ (100 μmol) for 8-h. The ratio of LC3-II to LC3-I and p62 to β-actin were shown at the bottom of each band (n = 3). **f** Representative fluorescence images of autophagosomes and autolysosomes in SMMC-7721 and Huh7 cells with PHF8 knockdown by tandem mCherry-GFP-LC3 fusion protein assay (magnification, × 400). **g** Quantification of autophagosomes and autolysosomes from random 5 high-power fields of the merged images of each group. * *P* < 0.05, ** *P* < 0.01, *** *P* < 0.001. Data were presented by mean ± SD

**Fig. 3:**
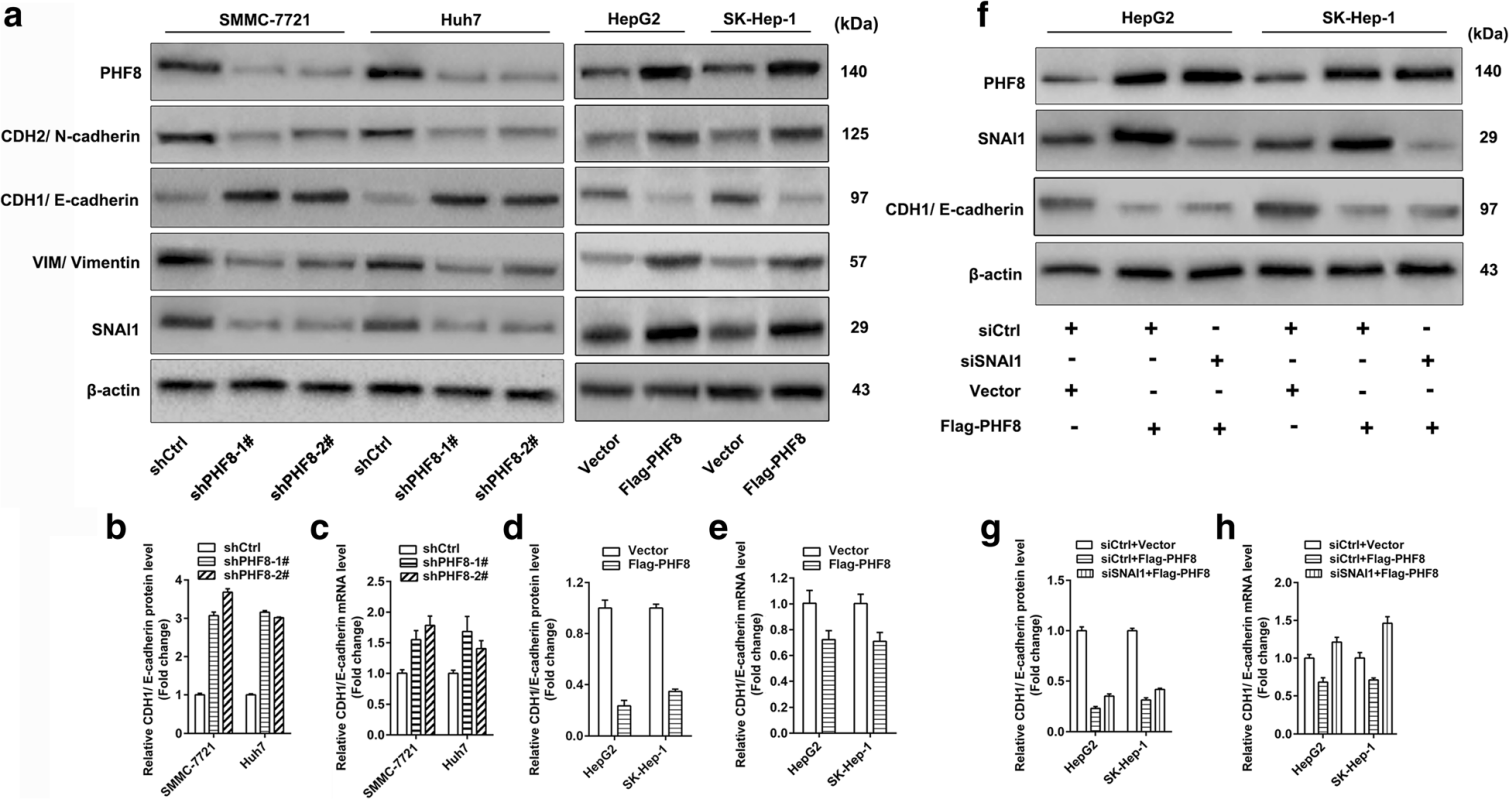
PHF8 regulates the expression of EMT markers. **a** Western-blot analysis of expression of SNAI1, VIM/ Vimentin, CDH2/ N-cadherin and E-cadherin. **b**, **d** quantification of E-cadherin protein amount. **c**, **e** qRT-PCR analysis of E-cadherin mRNA expression in SMMC-7721 and Huh7 cells with PHF8 knockdown and HepG2 and SK-Hep-1 cells with PHF8 exogenous overexpression. **f-h** Analysis of E-cadherin protein and mRNA expression after SNAI1 knockdown by siRNA in HepG2 and SK-Hep-1 cells with PHF8 overexpression. siCtrl was used for negative control. Data were presented by mean ± SD from three independent experiments

We next determined the potential biological functions of PHF8 in regulating malignant behaviors of HCC by RNA inference technology. SMMC-7721 and Huh7 cells were selected for transfection with scramble or PHF8-specific shRNAs because that they had highest expression of PHF8 among above cell lines (Fig. 1c and e). Inhibition efficiency of shRNAs was verified by qRT-PCR and immunoblotting assay (Fig. 2a). CCK8 results showed that PHF8 knockdown significantly impeded the proliferation of both cell lines (Fig. 2b). Furthermore, PHF8-silencing strikingly suppressed the migration and invasion as indicated by transwell migratory assay and Martrigel invasion assay, respectively (Fig. 2c and d), and regulated expression of EMT markers, including increased E-cadherin expression (epithelial marker) and reduced expression of SNAI1 (E-cadherin transcriptional repressor), VIM and N-cadherin (mesenchymal markers) (Fig. 3a). Reversely, PHF8 overexpression led to the opposite effects on proliferation, migration, invasion and expression of EMT markers in HepG2 and SK-Hep-1 cells (Fig. 3a and Additional file 7: Figure S2a to d), both of which had relative low expression of PHF8 (Fig. 1c and e). In view of transcriptional repression of E-cadherin by SNAI1, relative level of E-cadherin protein and mRNA were examined as well when PHF8 knockdown or overexpression (Fig. 3a-e). Of interest, abnormal PHF8 expression could induce more obvious change of E-cadherin protein expression than mRNA expression. In addition, SNAI1 knockdown significantly increased E-cadherin mRNA expression rather than protein expression in HepG2 and SK-Hep-1 cells with PHF8 overexpression. These data suggested that PHF8-mediated E-cadherin attenuation was not only dependent on SNAI upregulation. In vivo experiment proved that PHF8-silencing exhibited the slower tumor growth, smaller tumor size and less lung metastases compared with control group, indicating that PHF8-silencing blocked tumorigenesis and metastasis (Additional file 8: Figure S3). Taken together, PHF8 was able to promote tumorigenesis, tumor growth, EMT, migration, invasion and metastasis of HCC cells.

### PHF8 facilitates canonical autophagy elicited by starvation

Given that PHF8 is implicated in transcriptionally activating numerous genes and frequently upregulated by hypoxia, an important inducer of autophagy that could promotes tumorigenesis and metastasis of cancer cells [20, 21, 24, 25], we speculated that abnormal PHF8 expression was correlated with autophagy. The conversion of LC3B-I (cytosolic form) to LC3B-II (membrane-bound lipidated form) and degradation of SQSTM1/p62 serve as the index of the formation of autophagosome and autolysosome, respectively, and are widely measured by immunoblot assay to monitor autophagy [23]. Both production and clearance of autophagosomes regulate amount of LC3B-II and SQSTM1/p62 and could be distinguished when cells treated in starvation condition together with CQ, an inhibitor of fusion of autophagosome with lysosome by raising the lysosomal pH [23]. PHF8-silencing significantly inhibited LC3B-II transition in SMMC-771 and Huh7 cells under complete medium or EBSS starvation condition, with or without CQ (Fig. 2e). Moreover, PHF8-silencing suppressed the elimination of SQSTM1/p62 in both cells treated by starvation without CQ. These observations were confirmed by tandem mCherry-GFP-LC3 fluorescence microscopy assay. PHF8-silencing significantly reduced the numbers of both autophagosomes and autolysosomes (Fig. 2f and g), reflecting that PHF8 knockdown blocked the autophagosome biogenesis. These results were supported by the opposite biological phenomena in HepG2 and SK-Hep-1 cells with PHF8 overexpression (Additional file 7: Figure S2e to g). Overall, our data suggested that PHF8 contributed to autophagosome formation in HCC cells.

### PHF8 contributes to transcriptionally activating the expression of SNAI1, VIM and FIP200

**Fig. 4:**
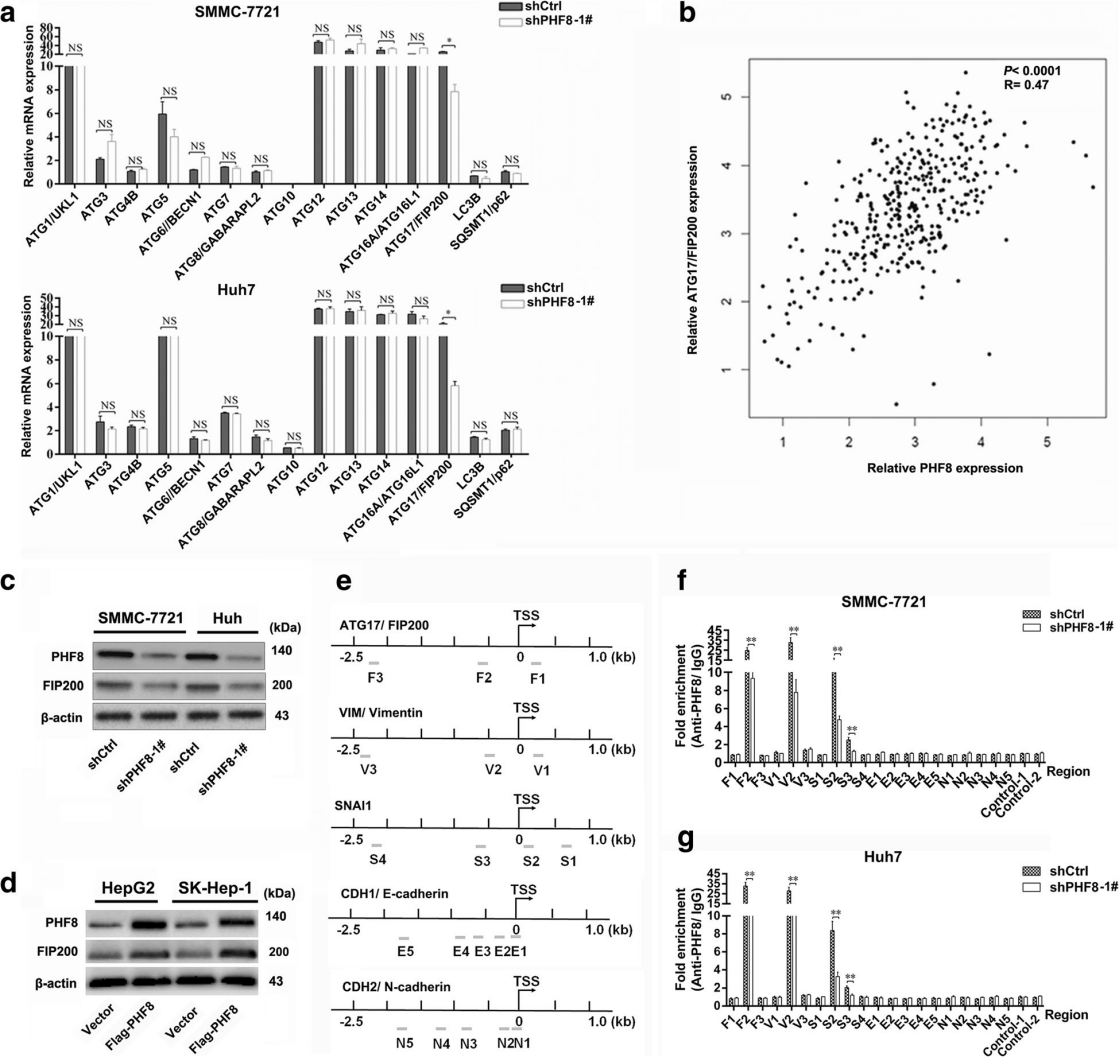
PHF8 is involved in transcriptional activation of FIP200, SNAI1 and VIM. **a** Effect of PHF8-silencing on mRNA expression of key autophagy-related genes. **b** The relationship between PHF8 expression and FIP200 expression by Gene Expression Profiling Interactive Analysis (GEPIA, http://gepia.cancer-pku.cn/). **c**, **d** Western-blot analysis of FIP200 expression in SMMC-7721 and Huh7 cells with PHF8 knockdown and HepG2 and SK-Hep-1 cells with PHF8 exogenous overexpression. **e** Schematic illustration of the sites of primer amplicons on promoter regions of FIP200, VIM, SNAI1, CDH1 and CDH2 for ChIP analysis. **f**, **g** Combining ChIP and qRT-PCR analysis to measure the levels of PHF8 presence at promoter of FIP200, VIM, SNAI1, CDH1 and CDH2 in SMMC-7721 and Huh7 cells with PHF8 knockdown. * *P* < 0.05, ** *P* < 0.01, NS, no significance. Data were presented by mean ± SD from three independent experiments

To explore the molecular mechanisms underlying PHF8-driven autophagy, we then analyzed mRNA expression profile of autophagy related genes (ATGs) and unveiled the decreased mRNA level of ATG17/ FIP200 by PHF8 knockdown (Fig. 4a). Intriguingly, the positive correlation of PHF8 expression with FIP200 expression was identified by Gene Expression Profiling Interactive Analysis (Fig. 4b) and substantiated by IHC study (Additional file 9: Table S6 and Additional file 10: Figure S4). Furthermore, immunoblotting assay confirmed that PHF8 positively regulated FIP200 expression (Fig. 4c and d). With a view to that PHF8 was a transcriptional co-activator [9, 29, 30], ChIP-qPCR assay was performed to investigate the molecular mechanism that PHF8 regulated the expression of EMT markers and FIP200, and results demonstrated that PHF8 was able to bind the promoter region of FIP200, SNAI1 and VIM, whereas failed to occupy the promoter site of N-cadherin and E-cadherin (Fig. 4f). These results suggested that PHF8 was involved in transcriptionally regulating the expression of FIP200, SNAI1 and VIM, rather than N-cadherin and E-cadherin.

### FIP200 overexpression reverses the inhibited effect of PHF8 knockdown on autophagy

**Fig. 5:**
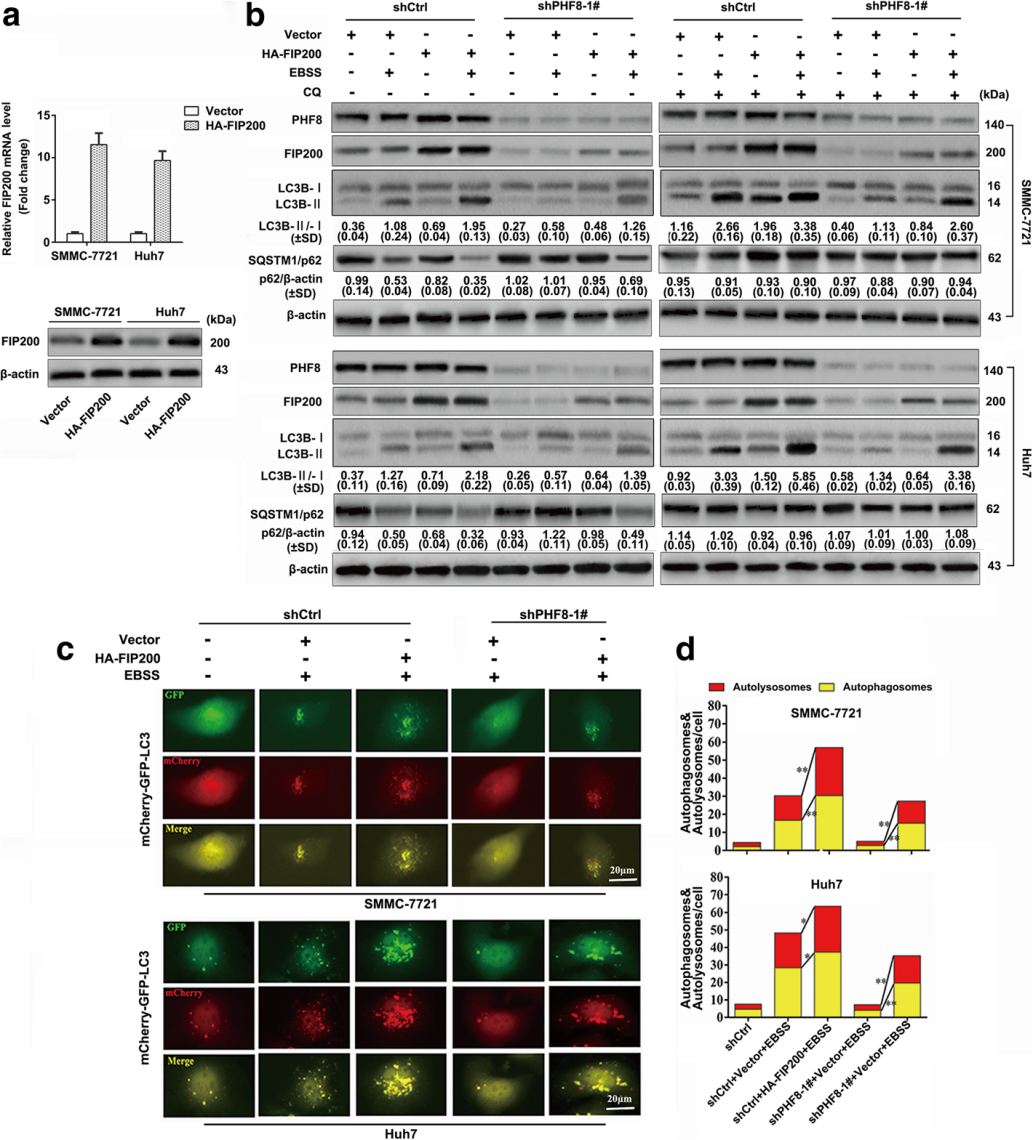
FIP200 of exogenous overexpression reverses the effect of PHF8-silencing on autophagy. **a** qRT-PCR and western-blot analysis of FIP200 expression in SMMC-7721 and Huh7 cells with exogenous overexpression of FIP200. Vector represented empty plasmid for negative control. **b** Representative immunoblot result of LC3B and p62 in SMMC-7721 and Huh7 cells with co-transfection of indicated shRNAs and plasmids (Vector or HA-FIP200) after cultured in complete medium with 10% FBS or EBSS starvation condition with or without CQ (100 μmol) for 8-h. **c** Representative fluorescence images of autophagosomes and autolysosomes in SMMC-7721 and Huh7 cells with co-transfection of shRNAs (shCtrl or shPHF8) and plasmids (Vector or HA-FIP200) (magnification, × 400). **d** Quantification of autophagosomes and autolysosomes from random 5 high-power fields of the merged images of each group. * *P* < 0.05, ** *P* < 0.01. Data were presented by mean ± SD from three independent experiments

Since FIP200 acts a key component involving in formation of autophagosome as well as the downstream gene of PHF8 [31, 32], we examined whether FIP200 was able to reverse the effect of PHF8-silencing on autophagy. As expected, exogenous overexpression of FIP200 promoted LC3B-II transition in SMMC-7721 and Huh7 cells with PHF8-silencing under complete medium or starvation condition in the presence or absence of CQ (Fig. 5a and b). Meanwhile, the amount of SQSMT1/p62 was significantly decreased in HCC cells with PHF8-silencing and FIP200 overexpression in the absence of CQ, although it kept stable in the presence of CQ. Furthermore, augmentation of FIP200 strikingly increased the number of autophagosomes and autolysosomes in both SMMC-7721 and Huh7 cells with or without PHF8 depletion (Fig. 5c and d), mirroring that FIP200 had the capacity of reversing the inhibited effect of PHF8-silencing on autophagy.

### PHF8-induced FIP200-dependent autophagy promotes degradation of E-cadherin, migration and invasion of HCC cells

**Fig. 6:**
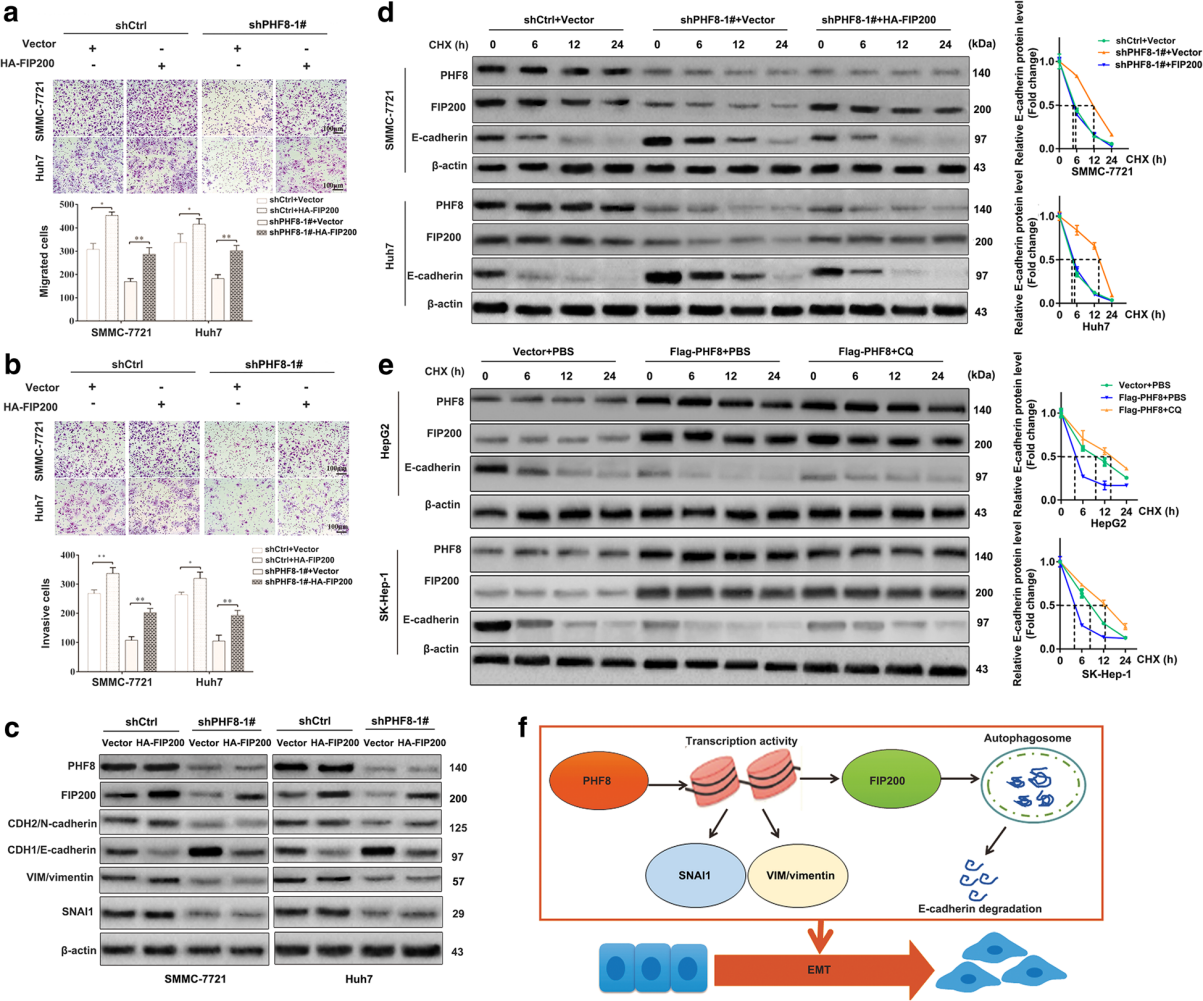
PHF8 promotes EMT and metastasis partly dependent on FIP200-mediated autophagic E-cadherin degradation. **a**, **b** Exogenous overexpression of FIP200 reverses effects of PHF8-knockdown on migration and invasion of SMMC-7721 and Huh7 cells (magnification, × 100). **c** Western-blot analysis of expression of FIP200 and EMT markers in SMMC-7721 and Huh7 cells with co-transfection of shPHF8–1# and HA-FIP200. **d**, **e** Western-blot analysis of E-cadherin expression in SMMC-7721 and Huh7 cells with co-transfection of shRNAs (shCtrl or shPHF8–1#) and plasmids (Vector or HA-FIP200), and in HepG2 and SK-Hep-1 cells transfected with PHF8 overexpression and pre-treated by CQ (100 μmol) for 12-h, and subsequently treated by CHX (20 μmol) for indicated time. The protein amount of PHF8 and FIP200 were measured as well. **f** Schematic representation of the mechanism of PHF8 promotes metastasis by transcriptional regulating expression of SNAI1 and VIM and FIP200-dependent autophagic degradation of E-cadherin. Data were presented as mean ± SD. * *P* < 0.05, ** *P* < 0.01

Now that autophagy process contributes to cancer metastasis by accelerating degradation of E-cadherin [20, 21], we presumed that PHF8-driven FIP200-dependent autophagy participated in metastasis and E-cadherin attenuation. Firstly, inhibition of autophagy process by CQ dramatically reduced the number of migrated and invasive HCC cells in SMMC-7721 and Huh7 cell lines (Additional file 11: Figure S5). Moreover, exogenous overexpression of FIP200 significantly increased the number of migrated and invasive cells and decreased E-cadherin amount, and similar results were observed after restoring FIP200 expression in PHF8-silencing context (Fig. 6a-c). Additionally, we demonstrated that E-cadherin degradation was delayed by PHF8-silencing and reaccelerated by exogenously increased expression of FIP200 in the presence of CHX (Fig. 6d). Reversely, E-cadherin degradation was accelerated by PHF8 overexpression whereas inhibited by CQ in HepG2 and SK-Hep-1 cells (Fig. 6e). These findings indicated that FIP200-dependent autophagy was a critical mechanism by which PHF8 suppressed E-cadherin and promoted migration and invasion in HCC.

## Discussion

PHF8 has been described as an oncoprotein that is positively associated with aggressive clinical features, poor prognosis and relapse of cancers [13, 16–18, 33]. Here, we confirmed the oncogenic role of PHF8, whose upregulation was significantly prevalent in HCC with more malignant phenotype and may serve as an independent risk factor for OS and RFS, despite no significant relation of PHF8 expression to OS from analysis of Protein Atlas Database (Additional file 12: Figure S6). Similar inconsistent observations exist in the studies of gastric cancer, prostate cancer and head and neck cancer [13, 17, 33]. This conflict is might be attributed to different study and statistical methods, sample size and population group enrolled. Another possibility is the discordant expression of protein and mRNA [34, 35], examined by IHC in the current and previous studies and RNA-Seq in Protein Atlas Database, respectively [13, 33], and consequently, statistical grouping was based on different criterion, the IHC-scoring in common for the former and the best separation or median line of mRNA expression for the latter. These results still provided an insight into predictive roles of PHF8 in prognosis, especially relapse of human cancers, although they need validation by large prospective studies.

PHF8 is ubiquitously expressed to target various genes in human cancers, and capable of pro-tumor and pro-metastasis [6–9, 12–18] . In accordance, our data showed that PHF8 positively regulated tumor proliferation, migration, invasion and metastasis of HCC cells in vitro and in vivo. More attention has been paid to the roles of PHF8 in promoting tumor metastasis and EMT by co-transcriptionally activating SNAI1 and VIM expression [16, 17], which was consistent with the current results. As a transmembrane protein that regulates cell adhesion and tumor metastasis, E-cadherin could be suppressed by PHF8 upregulation in other tumors [16, 17]. Accordantly, our data demonstrated that both mRNA and protein expression of E-cadherin were dramatically increased by PHF8 knockdown, whereas reduced by PHF8 overexpression in HCC. However, the discordant expression of mRNA and protein of E-cadherin were observed after PHF8 knockdown or overexpression. E-cadherin protein level was more susceptible to PHF8 abnormal expression than mRNA level. These phenomena could not be only addressed by SNAI1-mediated transcriptional repression of E-cadherin, because that PHF8 overexpression-mediated inhibition of E-cadherin protein expression, rather than mRNA expression, could not be completely abrogated by the SNAI1 knockdown. Moreover, PHF8 failed to bind the promoter region of E-cadherin, suggesting that PHF8 was unable to transcriptionally regulate E-cadherin expression. Therefore, PHF8-mediated attenuation of E-cadherin expression, especially protein level, was probably dependent on some indirect regulatory approaches including modulation of other molecules or post-translational regulation [16]. Although PHF8 upregulated N-cadherin, a key protein to facilitate transepithelial migration of tumor cells, much more effort is still under way to enucleate the exact mechanism of this process, on account of non-involvement of PHF8 in transcriptionally activating N-cadherin expression [16].

Increasing evidences have revealed that autophagy could accelerates metastasis of cancer cells [20, 21, 24, 25], and is regulated by histone methylation or demethylation [22, 36]. For instance, as a substrate of PHF8 [6, 7, 12], H3K9me2 blockage by histone methyltransferase inhibitor is able to significantly induces autophagy [37, 38]. Furthermore, PHF8 expression has been reported to be elevated under hypoxia stress circumstance which is in favor of autophagy activation [13, 36]. These observations are sufficient for us to infer the potential role of PHF8 in regulating autophagy, and our study provided the first evidence that PHF8 had the capacity of enhancing autophagy. In detail, PHF8-silencing blocked LC3B transition, the typical marker of autophagosome formation, and induced the accumulation of SQSTM1/ p62, which is negatively correlated with autophagic activity by immunoblotting assay [23]. In agreement with that, the number of both autophagosomes and autolysosomes were strikingly reduced by PHF8-silencing according to tandem mCherry-GFP-LC3 fluorescence assay. Opposite biological phenomena were generated by PHF8 overexpression. These data suggest that PHF8 is an important upstream regulator of initial step of autophagosome biogenesis. However, more effort is under way to provide additional evidences that PHF8 upregulation is associated with autophagy activation in HCC tissues by other assays, for instance, transmission electron microscopy and immunofluorescence assay.

According to the analysis of mRNA expression pattern of autophagy related genes and public database, PHF8 function was focused on increasing the expression of FIP200, an important protein interacts with ULK and ATG13 to generate a complex required for autophagosome formation [31, 32]. FIP200 inhibition mediated autophagy deficiency could suppress tumorigenesis [39]. In addition, ChIP-qPCR analysis exhibited that PHF8 could promote transcriptional activation of FIP200. Rescue experiment showed that FIP200 overexpression markedly reversed the effects of PHF8-silencing on migration, invasion and autophagy. Taking into consideration the promotion of PHF8 to autophagy and the implication of autophagy in E-cadherin degradation [20, 21], we could lift the veil of the indirect regulatory process through which PHF8 suppressed E-cadherin. FIP200-dependent autophagy activated by PHF8 was the crucial step for E-cadherin suppression, supported by more following observations. Firstly, E-cadherin degradation could be accelerated by PHF8 overexpression whereas delayed by PHF8-silencing when protein synthesis was inhibited by CHX. Secondly, the inhibition of autophagy by CQ was capable of blocking PHF8 overexpression-mediated degradation of E-cadherin. Thirdly, enforced expression of FIP200 was able to abrogate the inhibited effect of PHF8-silencing on E-cadherin degradation. Hence, we could safely concluded that PHF8-mediated FIP200-dependent autophagy was crucial for degradation of E-cadherin, EMT and tumor metastasis, and complementary to transcriptional repression of E-cadherin by SNAI1 upregulation in HCC.

## Conclusion

In summary, this study defined PHF8 as an oncoprotein that was related to aggressive phenotypes and may serve an independent predictor for OS and RFS in HCC. PHF8 was able to promote the autophagy, migration and invasion through upregulation of SNAI1, VIM, CDH2 and FIP200 and suppression of E-cadherin. Upregulation of PHF8 contributed to transcriptional activation of SNAI1, VIM and FIP200, and stimulated FIP200-dependent autophagy to accelerate E-cadherin degradation (Fig. 6f). Our findings provided a novel insight into function of PHF8 on tumor progression and metastasis, and suggested that PHF8 blockage might be a promising therapeutic approach for HCC.

## Additional files

Additional file 1: **Table S1.** Oligonucleotide sequences of primers for quantitative real time PCR. (DOCX 13 kb)

Additional file 2: **Table S2.** Details of primary antibodies. (DOCX 15 kb)

Additional file 3: **Figure S1.** Representative PHF8 IHC images with different stainingintensity. Magnification, × 40, × 200. (TIF 3414 kb)

Additional file 4: **Table S3.** Oligonucleotide sequences of primers for ChIP. (DOCX 16 kb)

Additional file 5: **Table S4.** Association of PHF8 expression with clinicopathologic features. (DOCX 17 kb)

Additional file 6: **Table S5.** Univariate- and Multivariate- analysis of risk factors for relapse-free survival (RFS) and overall survival (OS). (DOCX 18 kb)

Additional file 7: **Figure S2.** Exogenous overexpression of PHF8 enhances proliferation, migration, invasion and autophagy of HepG2 and SK-Hep-1 cells in vitro. **a** qRT-PCR and western-blot analysis of transfection efficiency of Flag-PHF8 plasmid in HepG2 and SK-Hep-1 cells. Empty plasmid (Vector) was used for negative control. **b** Enhanced proliferation of HepG2 and SK-Hep-1 cells in PHF8 overexpression group by CCK8 assasy (*n* = 6). **c**, **d** Representative images and quantification of migrated and invasive cells by transwell assay in HepG2 and SK-Hep-1 cells (*n* = 3, magnification, × 100). **e** Representative immunoblot results of LC3B and p62 in HepG2 and SK-Hep-1 cells transfected with indicated plasmids, and then cultured in complete medium with 10% FBS or EBSS starvation condition with or without CQ (100 μmol) for 8-h (*n* = 3). **f** Representative fluorescence images of autophagosomes and autolysosomes in HepG2 and SK-Hep-1 cells with PHF8 overexpression by tandem mCherry-GFP-LC3 fusion protein assay (magnification, × 400). **g** Quantification of autophagosomes and autolysosomes from random 5 high-power fields of the merged images of each group. * *p* < 0.05, ** *P* < 0.01, *** *P* < 0.001. Data were presented by mean ± SD. (TIF 6912 kb)

Additional file 8: **Figure S3.** The blockage of PHF8 inhibits tumorigenesis and metastasis in vivo. **a – d** Appearance of primary tumor, tumor growth curves and tumor weight in two groups (*n* = 6). **d** Overview of lung metastatic lesions (upper panel, white arrow indicated the metastatic colonization) and HE images (lower panel, magnification, × 100). **e** The number of lung metastatic nets of each group was counted in a low power field (*n* = 6). * *P* < 0.05, ** *P* < 0.01, *** *P* < 0.001. Data were presented by mean ± SD. (TIF 5523 kb)

Additional file 9: **Table S6.** Correlation of PHF8 expression with expression of ATG17/ FIP200 and E-cadherin based on immunohistochemistry analysis. (DOCX 14 kb)

Additional file 10: **Figure S4.** Representative images of HE and IHC staining of HCC tissues with or without vascular invasion. IHC staining for PHF8, FIP200 and E-cadherin. Magnification, × 40 and × 200. (TIF 6378 kb)

Additional file 11: **Figure S5.** CQ blocks the migration and invasion of HCC cells. **a**, **b** SMMC-7721 and Huh7 cells pre-treated by CQ (100 μmol) for 12-h were subjected to transwell migration or invasion assay. Magnification, × 100. *** *P* < 0.001. Data were presented by mean ± SD. (TIF 2688 kb)

Additional file 12: **Figure S6.** Relationship between PHF8 expression and overall survival of human cancers from Protein Atlas Database (https://www.proteinatlas.org/). Patients were divided into two groups by the line of best separation of mRNA expression. (TIF 3076 kb)

## Abbreviations

95% CI: 95% confidence interval
AFP: Alpha fetoprotein
AJCC: American Joint Committee on Cancer
ATG: Autophagy related gene
CCK8: Cell Count Kit 8
ChIP: Chromatin immunoprecipitation
CHX: Cycloheximide
CQ: Chloroquine
EMT: Epithelial-mesenchymal transition
GEO: Gene expression omnibus
H&E: Hematoxylin and eosin
HBsAg: Hepatitis B surface antigen
HCC: Hepatocellular carcinoma
HR: Hazard ratio
IHC: Immunohistochemistry
OS: Overall survival
PHF8: Plant homeodomain finger protein 8
RFS: Relapse free survival
