# Correction to: PHF8 upregulation contributes to autophagic degradation of E-cadherin, epithelial-mesenchymal transition and metastasis in hepatocellular carcinoma

**DOI:** 10.1186/s13046-018-0944-7

**Published:** 2018-11-07

**Authors:** Wuhua Zhou, Li Gong, Qinchuan Wu, Chunyang Xing, Bajin Wei, Tianchi Chen, Yuan Zhou, Shengyong Yin, Bin Jiang, Haiyang Xie, Lin Zhou, Shusen Zheng

**Affiliations:** 1 Division of Hepatobiliary and Pancreatic Surgery, Department of Surgery, The First Affiliated Hospital, Zhejiang University, Hangzhou, China; 2 NHFPC Key Laboratory of Combined Multi-Organ Transplantation, Hangzhou, China; 3 Key Laboratory of the Diagnosis and Treatment of Organ transplantation, CAMS, Hangzhou, China; 4 Key Laboratory of Organ Transplantation, Hangzhou, Zhejiang Province China; 5 Collaborative Innovation Center for Diagnosis Treatment of Infectious Disease, Zhejiang University, Hangzhou, China; 6 Department of Hepatobiliary and Pancreatic Surgery, Taihe Hospital, Shiyan, China; 7 Department of Endocrinology, Taihe Hospital, Shiyan, China

## Correction

In the publication of this article [1], there are two inadvertent errors.

The first error is that the minus signals representing no addition of CQ are duplicated in the second half of both left and right panel of Fig. 2e**,** and these minus signals should be plus signals.

**Fig. 2:**
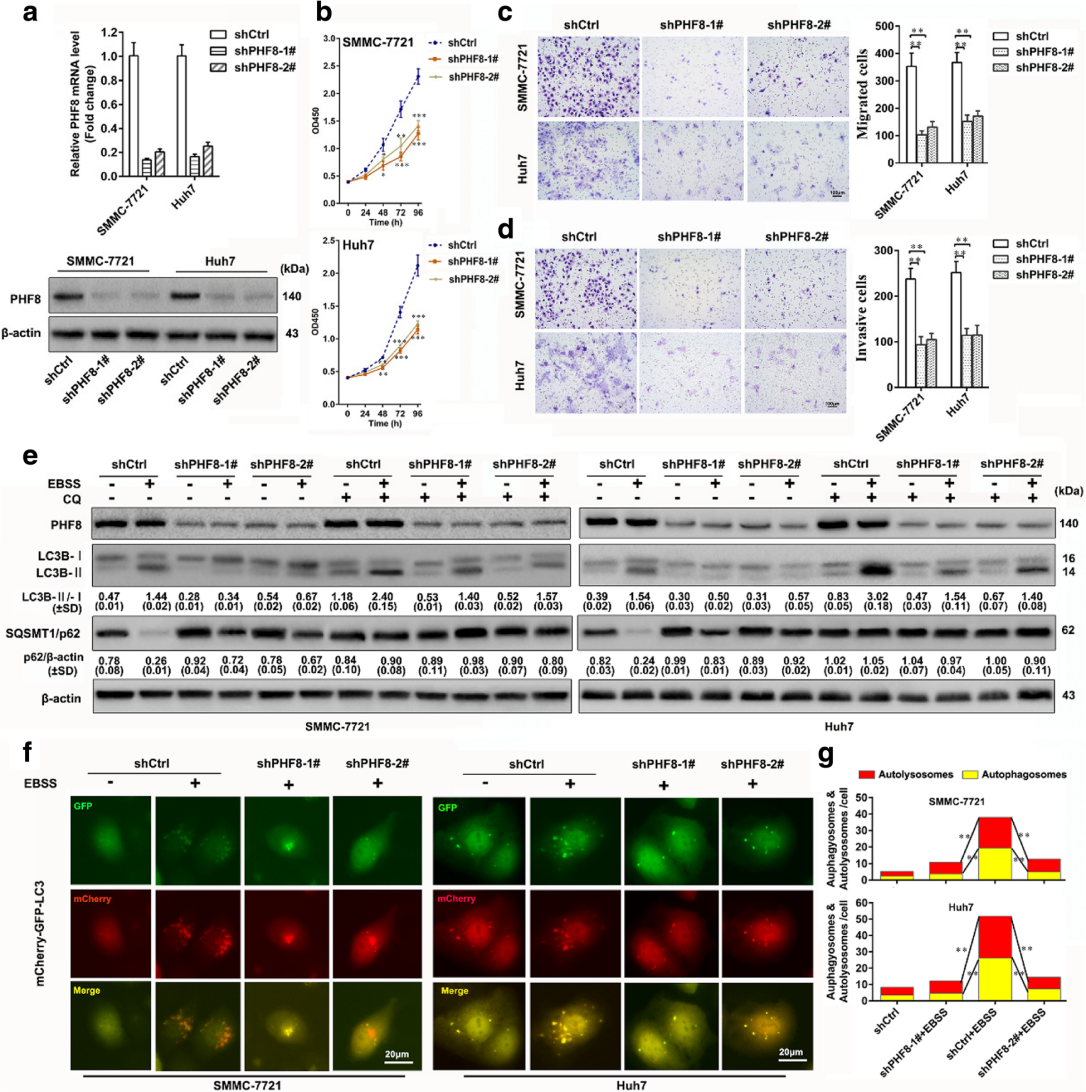
PHF8 knockdown significantly suppresses proliferation, migration, invasion and autophagy of HCC cells in vitro. **a** Determination of transfection efficiency of shRNAs targeting PHF8 in SMMC-7721 and Huh7 by qRT-PCR and western-blot assay. Scramble shRNA (shCtrl) was used for negative control. **b** Inhibited proliferation of SMMC-7721 and Huh7 cells in PHF8 knockdown group by CCK8 assasy (*n* = 6). **c**, **d**) Representative images and quantification of migrated and invasive cells by transwell assay in SMMC-7721 and Huh7 cells (*n* = 3, magnification, × 100). **e** Representative immunoblot results of autophagy markers, LC3B and p62 in SMMC-7721 and Huh7 cells with PHF8 knockdown. Both cell lines transfected with indicated shRNAs were cultured in complete medium with 10% FBS or EBSS starvation condition with or without CQ (100 μmol) for 8-h. The ratio of LC3-II to LC3-I and p62 to β-actin were shown at the bottom of each band (*n* = 3). **f** Representative fluorescence images of autophagosomes and autolysosomes in SMMC-7721 and Huh7 cells with PHF8 knockdown by tandem mCherry-GFP-LC3 fusion protein assay (magnification, × 400). **g** Quantification of autophagosomes and autolysosomes from random 5 high-power fields of the merged images of each group. * *P* < 0.05, ** *P* < 0.01, *** *P* < 0.001. Data were presented by mean ± SD

The second error is that a letter “m” is missed before the last letter “g” in the text along the vertical axis of Additional file 8: Figure S3c as “Tumor weight (**g**)”.

It should instead read: “Tumor weight (**mg**)”.

These errors do not affect discussions and conclusions drawn in the article.

Figure 2 with corrected Fig. 2e and Additional file 8: Figure S3 with corrected Figure S3c are included in this correction and shown hereafter.

## Additional file

Additional file 8: **Figure S3.** The blockage of PHF8 inhibits tumorigenesis and metastasis in vivo. a – d) Appearance of primary tumor, tumor growth curves and tumor weight in two groups (*n* = 6). d Overview of lung metastatic lesions (upper panel, white arrow indicated the metastatic colonization) and HE images (lower panel, magnification, × 100). e The number of lung metastatic nets of each group was counted in a low power field (*n* = 6). * *P* < 0.05, ** *P* < 0.01, *** *P* < 0.001. Data were presented by mean ± SD. (DOCX 956 kb)
