# Correction to: PHF8 upregulation contributes to autophagic degradation of E-cadherin, epithelial-mesenchymal transition and metastasis in hepatocellular carcinoma

**DOI:** 10.1186/s13046-019-1452-0

**Published:** 2019-10-31

**Authors:** Wuhua Zhou, Li Gong, Qinchuan Wu, Chunyang Xing, Bajin Wei, Tianchi Chen, Yuan Zhou, Shengyong Yin, Bin Jiang, Haiyang Xie, Lin Zhou, Shusen Zheng

**Affiliations:** 1 Division of Hepatobiliary and Pancreatic Surgery, Department of Surgery, The First Affiliated Hospital, School of Medicine, Zhejiang University, Hangzhou, China; 2 NHFPC Key Laboratory of Combined Multi-Organ Transplantation, Hangzhou, China; 3 Key Laboratory of the Diagnosis and Treatment of Organ transplantation, CAMS, Hangzhou, China; 4 Key Laboratory of Organ Transplantation, Hangzhou, Zhejiang Province China; 5 Collaborative Innovation Center for Diagnosis Treatment of Infectious Disease, Zhejiang University, Hangzhou, China; 6 Department of Hepatobiliary and Pancreatic Surgery, Taihe Hospital, Shiyan, China; 7 Department of Endocrinology, Taihe Hospital, Shiyan, China

**Correction to: J Exp Clin Cancer Res (2018) 37:215**

**https://doi.org/10.1186/s13046-018-0890-4**

In the publication of our publication [1], we have noticed there is a wrong label in Fig. 1e, in which the position of “HCC” and “Adjacent” should be transposed. Actually, this error does not affect discussions and conclusions of the original article. Fig. 1 with corrected labels of Fig. 1e is shown below:

**Fig. 1:**
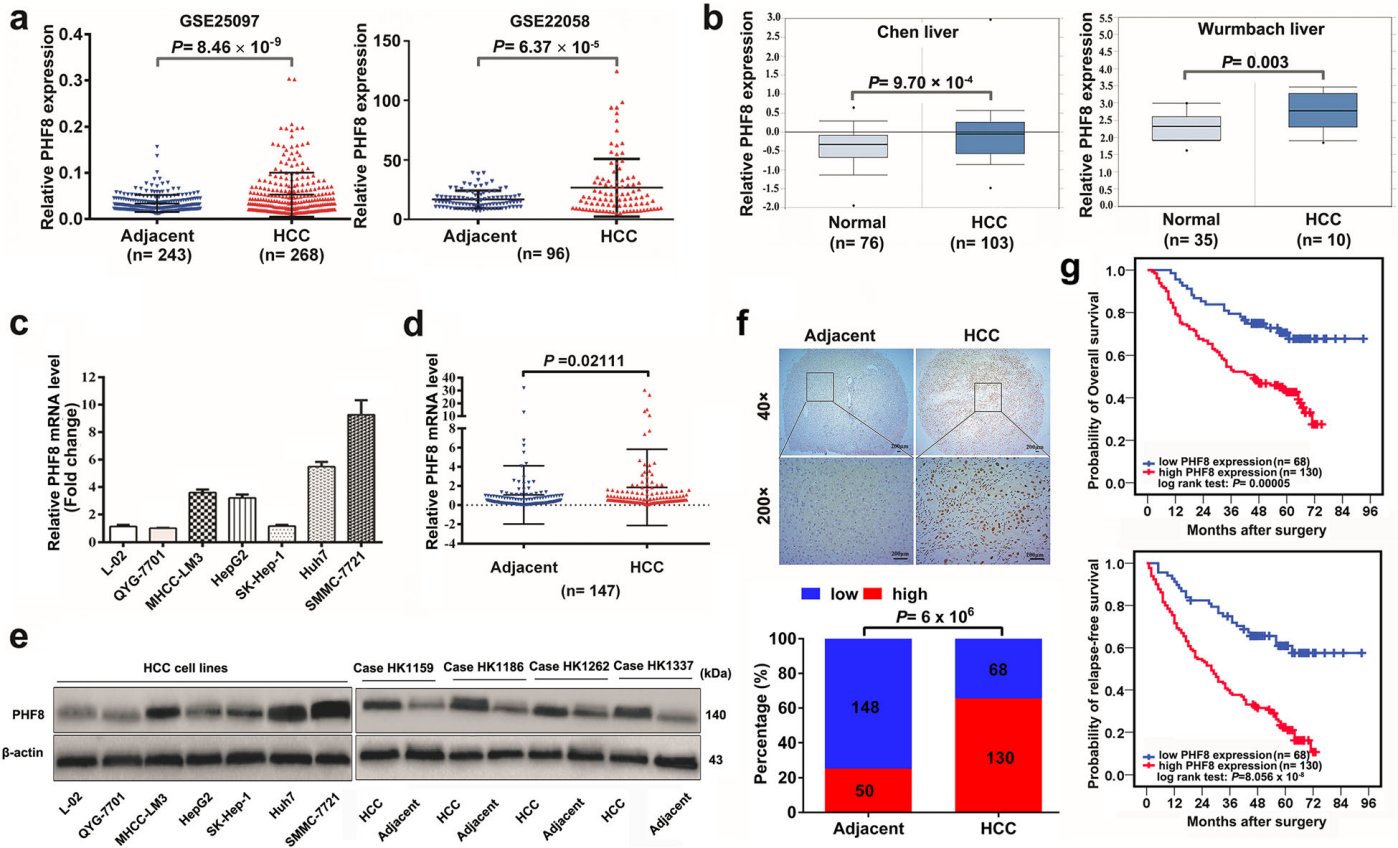
.
