# Supplementary material for: PHF8 upregulation contributes to autophagic degradation of E-cadherin, epithelial-mesenchymal transition and metastasis in hepatocellular carcinoma
Source: J Exp Clin Cancer Res. 2018 Sep 4;37:215. doi: 10.1186/s13046-018-0890-4 (PMC6122561; doi:10.1186/s13046-018-0890-4)
Supplement: Supplementary file 1 — Table S1. Oligonucleotide sequences of primers for quantitative real time PCR. (DOCX 13 kb) [file 13046_2018_890_MOESM1_ESM.docx]

Table S1. Oligonucleotide sequences of primers for quantitative real time PCR

| Gene symbol | Sense (5' -> 3') | Antisense (5' -> 3') |
| --- | --- | --- |
| ULK1/ATG1 | GGCAAGTTCGAGTTCTCCCG | CGACCTCCAAATCGTGCTTCT |
| ATG3 | GACCCCGGTCCTCAAGGAA | TGTAGCCCATTGCCATGTTGG |
| ATG4B | ATGGACGCAGCTACTCTGAC | TTTTCTACCCAGTATCCAAACGG |
| MAP1LC3B/LC3B | TACGAGCAGGAGAAAGACGAGG | GGCAGAGTAGGTGGGTTGGTG |
| ATG5 | AAAGATGTGCTTCGAGATGTGT | CACTTTGTCAGTTACCAACGTCA |
| BECN1/ATG6 | CCATGCAGGTGAGCTTCGT | GAATCTGCGAGAGACACCATC |
| ATG7 | CAGTTTGCCCCTTTTAGTAGTGC | CCAGCCGATACTCGTTCAGC |
| GABARAPL2/ATG8 | ACTCGCTGGAACACAGATGC | TCTGAGAGCCTGAGACCTTTT |
| ATG10 | AGACCATCAAAGGACTGTTCTGA | GGGTAGATGCTCCTAGATGTGAC |
| ATG12 | CTGCTGGCGACACCAAGAAA | CGTGTTCGCTCTACTGCCC |
| ATG13 | TTGCTATAACTAGGGTGACACCA | CCCAACACGAACTGTCTGGA |
| ATG14 | GCGCCAAATGCGTTCAGAG | AGTCGGCTTAACCTTTCCTTCT |
| ATG16L1/ATG16A | AACGCTGTGCAGTTCAGTCC | AGCTGCTAAGAGGTAAGATCCA |
| ATG17/FIP200 | AATGCCGTCAAACTATTGCCA | GGGCGTAGAGCCGATCTTC |
| SQSTM1/p62 | GCACCCCAATGTGATCTGC | CGCTACACAAGTCGTAGTCTGG |
| ACTB/β-actin | TGACAGGATGCAGAAGGAGA | GCTGGAAGGTGGACAGTGAG |
