# Supplementary material for: PHF8 upregulation contributes to autophagic degradation of E-cadherin, epithelial-mesenchymal transition and metastasis in hepatocellular carcinoma
Source: J Exp Clin Cancer Res. 2018 Sep 4;37:215. doi: 10.1186/s13046-018-0890-4 (PMC6122561; doi:10.1186/s13046-018-0890-4)
Supplement: Supplementary file 2 — Table S2. Details of primary antibodies. (DOCX 15 kb) [file 13046_2018_890_MOESM2_ESM.docx]

Table S2. Details of primary antibodies

| Antibody | Origin | Assay | Dilution |
| --- | --- | --- | --- |
| PHF8 | #ab36068 Abcam | WB | 1:1000 |
| ATG17/ FIP200 | #ab176816 Abcam | WB | 1:2500 |
| SQSTM1/p62 | #ab56416 Abcam | WB | 1:2000 |
| LC3B | #ab51520 Abcam | WB | 1:3000 |
| CDH2/ N-cadherin | #ab18203 Abcam | WB | 1:1000 |
| CDH1/ E-cadherin | #ab219332 Abcam | WB | 1:500 |
| VIM/ Vimentin | #5741 CST | WB | 1:1000 |
| SNAI1 | # ab216347 Abcam | WB | 1:500 |
| β-actin | #A5441 Sigma-Aldrich | WB | 1:4000 |
| PHF8 | #ab36068 Abcam | IHC | 1:150 |
| ATG17/ FIP200 | #ab185346 Abcam | IHC | 1:200 |
| CDH1/ E-cadherin | # ab219332 Abcam | IHC | 1:150 |
