# Supplementary figures and images for: PHF8 upregulation contributes to autophagic degradation of E-cadherin, epithelial-mesenchymal transition and metastasis in hepatocellular carcinoma
Source: J Exp Clin Cancer Res. 2018 Sep 4;37:215. doi: 10.1186/s13046-018-0890-4 (PMC6122561; doi:10.1186/s13046-018-0890-4)

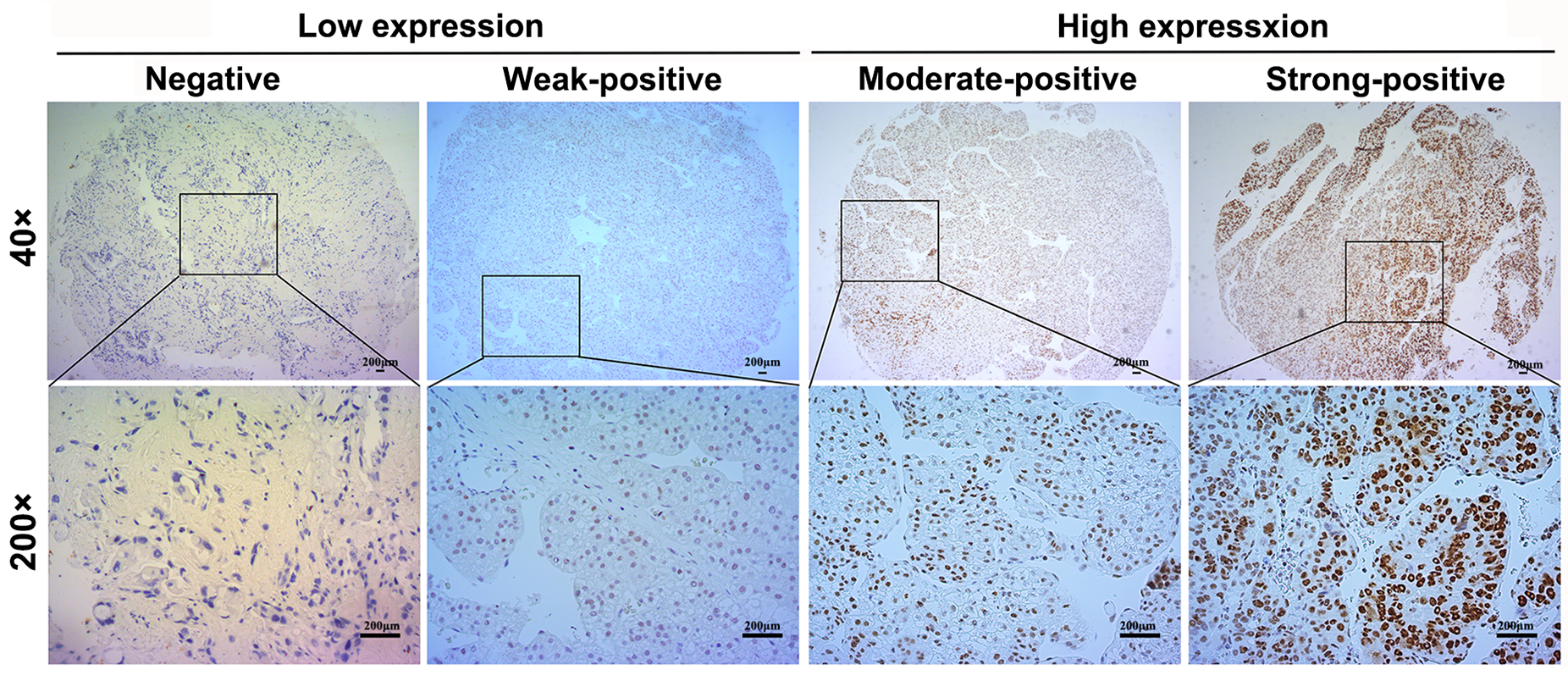

Supplement: Supplementary file 3 — Figure S1. Representative PHF8 IHC images with different stainingintensity. Magnification, × 40, × 200. (TIF 3414 kb) [file 13046_2018_890_MOESM3_ESM.tif]

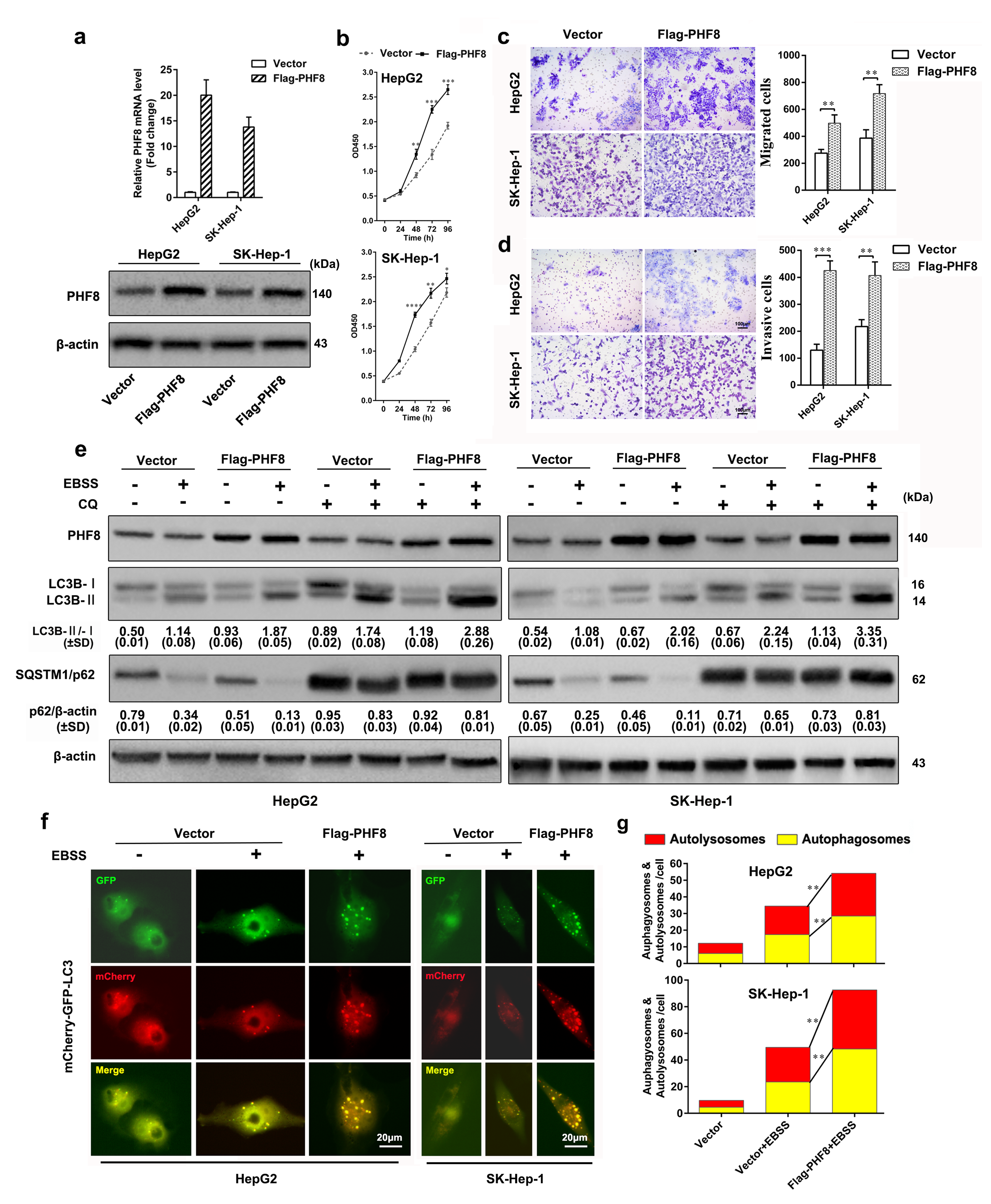

Supplement: Supplementary file 7 — Figure S2. Exogenous overexpression of PHF8 enhances proliferation, migration, invasion and autophagy of HepG2 and SK-Hep-1 cells in vitro. a qRT-PCR and western-blot analysis of transfection efficiency of Flag-PHF8 plasmid in HepG2 and SK-Hep-1 cells. Empty plasmid (Vector) was used for negative control. b Enhanced proliferation of HepG2 and SK-Hep-1 cells in PHF8 overexpression group by CCK8 assasy (n = 6). c, d Representative images and quantification of migrated and invasive cells by transwell assay in HepG2 and SK-Hep-1 cells (n = 3, magnification, × 100). e Representative immunoblot results of LC3B and p62 in HepG2 and SK-Hep-1 cells transfected with indicated plasmids, and then cultured in complete medium with 10% FBS or EBSS starvation condition with or without CQ (100 μmol) for 8-h (n = 3). f Representative fluorescence images of autophagosomes and autolysosomes in HepG2 and SK-Hep-1 cells with PHF8 overexpression by tandem mCherry-GFP-LC3 fusion protein assay (magnification, × 400). g Quantification of autophagosomes and autolysosomes from random 5 high-power fields of the merged images of each group. * p < 0.05, ** P < 0.01, *** P < 0.001. Data were presented by mean ± SD. (TIF 6912 kb) [file 13046_2018_890_MOESM7_ESM.tif]

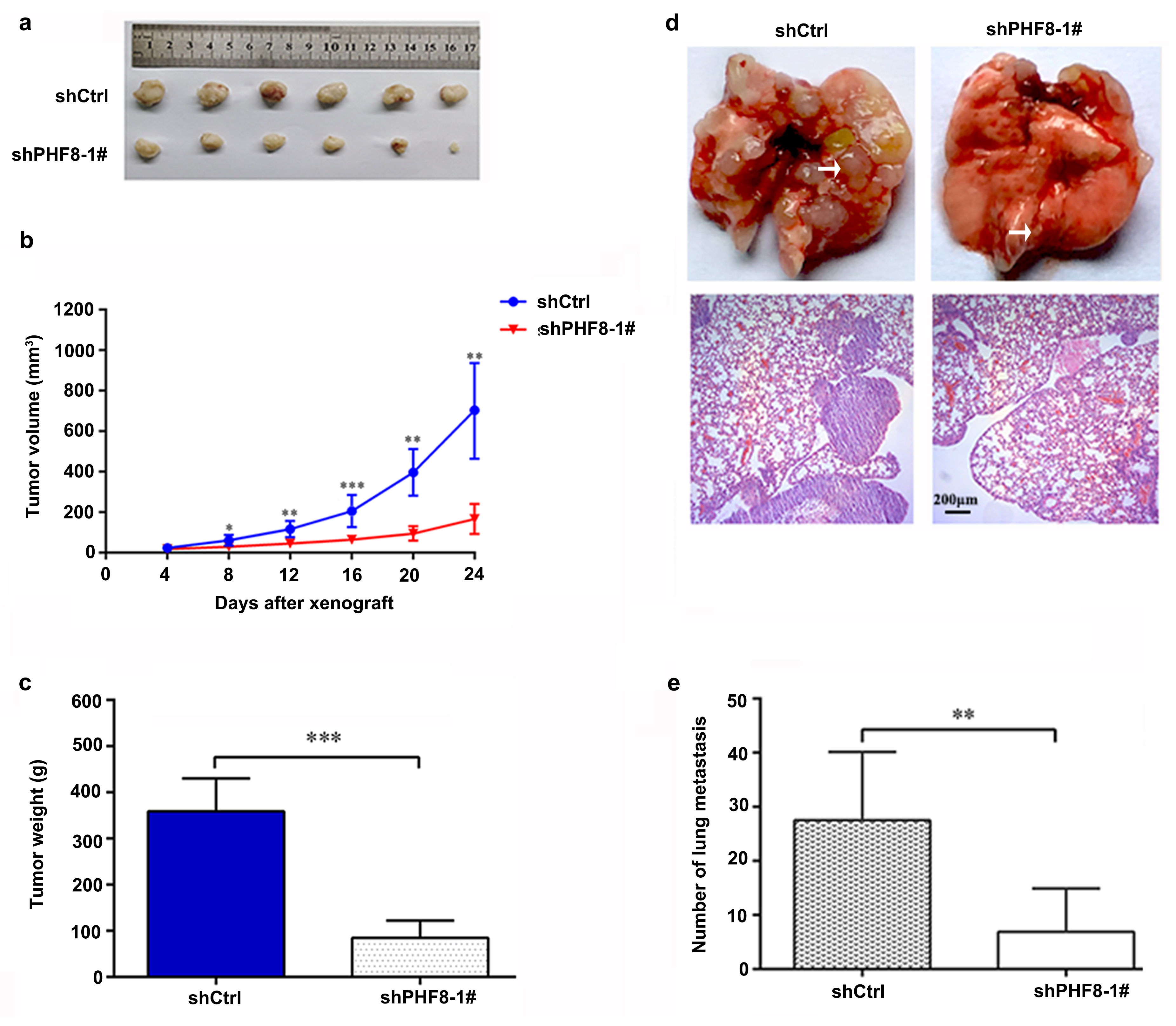

Supplement: Supplementary file 8 — Figure S3. The blockage of PHF8 inhibits tumorigenesis and metastasis in vivo. a – d Appearance of primary tumor, tumor growth curves and tumor weight in two groups (n = 6). d Overview of lung metastatic lesions (upper panel, white arrow indicated the metastatic colonization) and HE images (lower panel, magnification, × 100). e The number of lung metastatic nets of each group was counted in a low power field (n = 6). * P < 0.05, ** P < 0.01, *** P < 0.001. Data were presented by mean ± SD. (TIF 5523 kb) [file 13046_2018_890_MOESM8_ESM.tif]

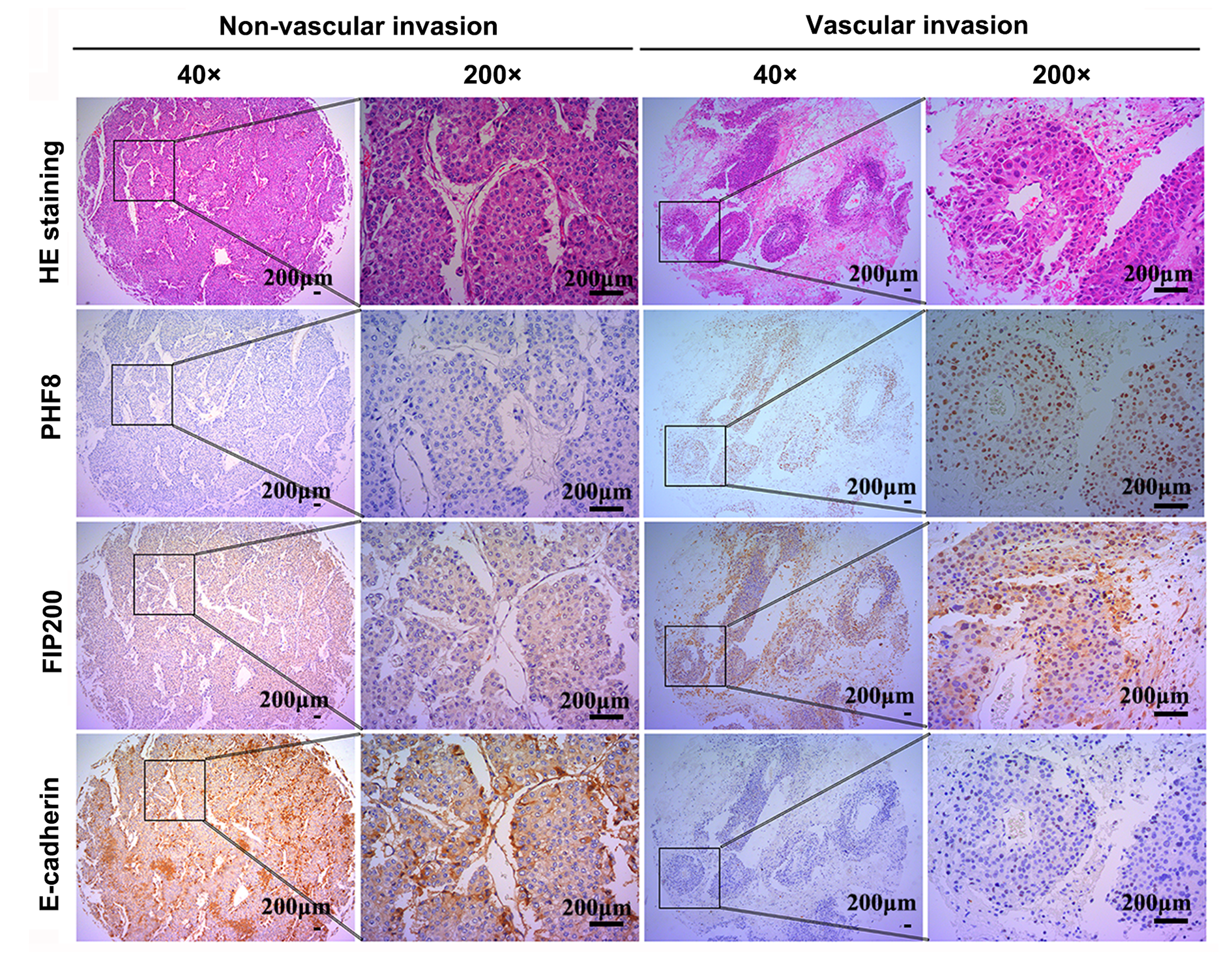

Supplement: Supplementary file 10 — Figure S4. Representative images of HE and IHC staining of HCC tissues with or without vascular invasion. IHC staining for PHF8, FIP200 and E-cadherin. Magnification, × 40 and × 200. (TIF 6378 kb) [file 13046_2018_890_MOESM10_ESM.tif]

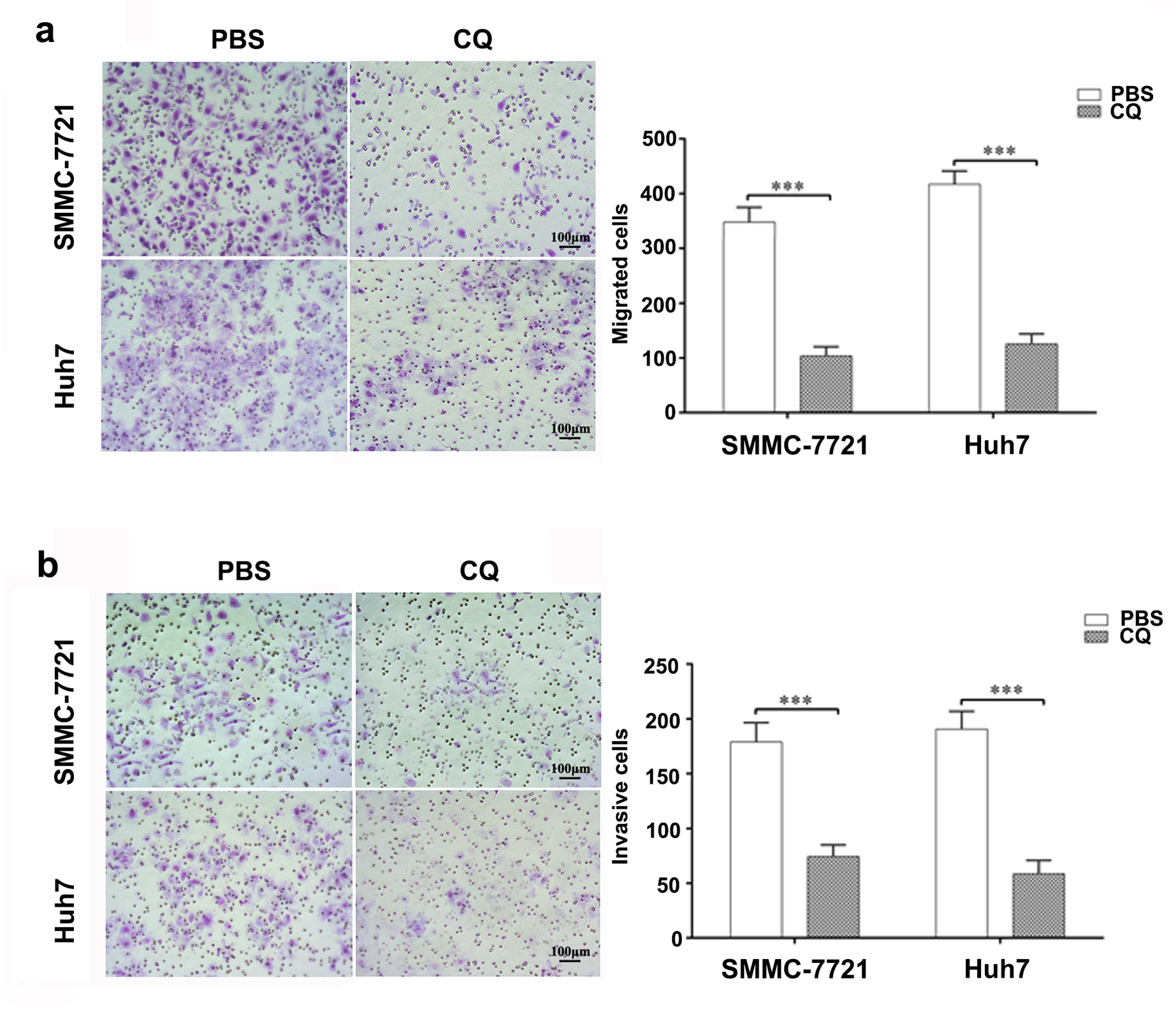

Supplement: Supplementary file 11 — Figure S5. CQ blocks the migration and invasion of HCC cells. a, b SMMC-7721 and Huh7 cells pre-treated by CQ (100 μmol) for 12-h were subjected to transwell migration or invasion assay. Magnification, × 100. *** P < 0.001. Data were presented by mean ± SD. (TIF 2688 kb) [file 13046_2018_890_MOESM11_ESM.tif]

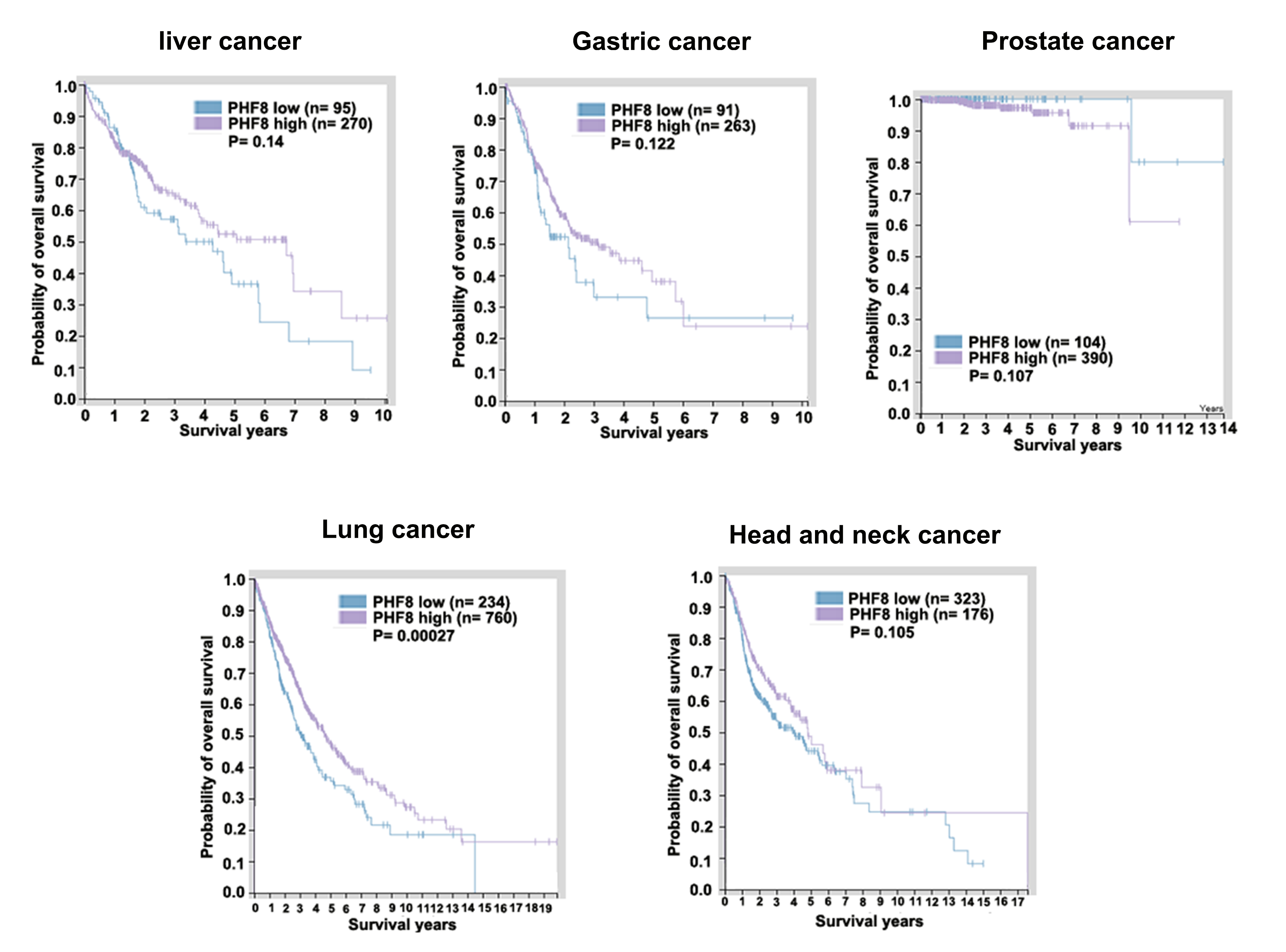

Supplement: Supplementary file 12 — Figure S6. Relationship between PHF8 expression and overall survival of human cancers from Protein Atlas Database (https://www.proteinatlas.org/). Patients were divided into two groups by the line of best separation of mRNA expression. (TIF 3076 kb) [file 13046_2018_890_MOESM12_ESM.tif]
