# Supplementary material for: PHF8 upregulation contributes to autophagic degradation of E-cadherin, epithelial-mesenchymal transition and metastasis in hepatocellular carcinoma
Source: J Exp Clin Cancer Res. 2018 Sep 4;37:215. doi: 10.1186/s13046-018-0890-4 (PMC6122561; doi:10.1186/s13046-018-0890-4)
Supplement: Supplementary file 4 — Table S3. Oligonucleotide sequences of primers for ChIP. (DOCX 16 kb) [file 13046_2018_890_MOESM4_ESM.docx]

Table S3. Oligonucleotide sequences of primers for ChIP

| Gene symbol | Primers of  target regions | Sense (5' -> 3') | Antisense (5' -> 3') |
| --- | --- | --- | --- |
| FIP200/ATG17 | F1 (211~304) | TCCCTGCCTCCTAGAGTT | GTTACCAACCGCCCATTC |
|  | F2 (-599~-517) | GGTTGTGGCGTTTACATTT | AGACCAGGTAAGGCAAGA |
|  | F3 (-2292~-2213) | TGGAGGTCCTTGTTAATGAT | AGTCCTCAGTCCAATCTCA |
| VIM/vimentin | V1 (203~267) | GTAGCACTGAGAACTAGCA | ACCGTTAGACCAGATTGATT |
|  | V2 (-531~-472) | AGGTTAAGGTGACCGAATC | GGACAGCAATGCACAGTA |
|  | V3 (-2406~-2347) | CCCTCCTTCTTCTTTCTAACA | GCCAAGAGAACAGCATCTA |
| SNAI1 | S1 (633~728) | AATGACCTCCTTCAACTGG | AATTATCCACAGGACAGACC |
|  | S2 (23~109) | CTAGCGAGTGGTTCTTCTG | TCCTGACGAGGAAAGAGC |
|  | S3 (-690~-592) | GGCTCTGAGTGTTCTGTC | ACCCGTTCCTTCCCTTAT |
|  | S4 (-2176~-2078) | CAGTGAGCCGTGATTGTG | TTATACGAGGTTCTGAAGCA |
| CDH1/E-cadherin | E1 (-67~30) | CTCAGCCAATCAGCGGTA | ACAGGTGCTTTGCAGTTC |
|  | E2 (-274~-209) | TCTGATCCCAGGTCTTAGT | ACGGTTCTGATTCCACTG |
|  | E3 (-636~-536) | CTGAAATCCTAGCACTTTGG | GCTGTGTCTCCCTGTATTG |
|  | E4 (-963~-901) | GCCTGTAATCCAACACTTC | TCTGGAACTCCTGACCTC |
|  | E5 (-1718-~1645) | AGAGCTGGTCAGTGTCAA | AGTAACAATAGGTGCTGGAC |
| CDH2/N-cadherin | N1 (-71~3) | AAACCACGAGCCCGAAAC | AGGATGTGGAGGTGGAAGT |
|  | N2 (-244~-168) | TGTTCCAGTACATCCTCAAG | GGCAGACACAGCAAACTA |
|  | N4 (-871~-812) | TCACCATTCTTCATCTCCATTAG | ATTGGTAGCAGGTTCATCTT |
|  | N4 (-1293~-1227) | GGTGGTAGGAGAGGGTAAA | CCTGTTAGTGGTACTGTTCA |
|  | N5 (-1728~-1655) | TGATCGGTATCTTGAAACTC | CTGCCTTTATTGCTTGCT |
| negative control | Control-1 | CGAAGGTTACGAACAGGAT | TCTATCTGTGGTGCCATCTA |
|  | Control-2 | TCTCCTCTCTAGCATACCTATA | TGTTCCTTCTCCAGCATATC |
