# Supplementary material for: PHF8 upregulation contributes to autophagic degradation of E-cadherin, epithelial-mesenchymal transition and metastasis in hepatocellular carcinoma
Source: J Exp Clin Cancer Res. 2018 Sep 4;37:215. doi: 10.1186/s13046-018-0890-4 (PMC6122561; doi:10.1186/s13046-018-0890-4)
Supplement: Supplementary file 5 — Table S4. Association of PHF8 expression with clinicopathologic features. (DOCX 17 kb) [file 13046_2018_890_MOESM5_ESM.docx]

Table S4. Association of PHF8 expression with clinicopathologic features

| Clinicopathologic variable | N | PHF8 expression | | *P* value |
| --- | --- | --- | --- | --- |
|  |  | high | low |  |
| Gender  male  female |  |  |  |  |
|  | 177 | 115 | 62 | 0.556 |
|  | 21 | 15 | 6 |  |
| Age, y  ≤ 50  > 50 |  |  |  |  |
|  | 60 | 39 | 21 | 0.898 |
|  | 138 | 91 | 47 |  |
| Cirrhosis  no  yes |  |  |  |  |
|  | 34 | 20 | 14 | 0.357 |
|  | 164 | 110 | 54 |  |
| HBsAg^b^  negative  positive |  |  |  |  |
|  | 32 | 19 | 13 | 0.427 |
|  | 165 | 110 | 55 |  |
| Preoperative AFP, ng/mL  ≤ 20  > 20 |  |  |  |  |
|  | 73 | 43 | 30 | 0.126 |
|  | 125 | 87 | 38 |  |
| Tumor number  single  multiple (≥2) |  |  |  |  |
|  | 144 | 92 | 52 | 0.392 |
|  | 54 | 38 | 16 |  |
| Maximal tumor size, cm  ≤ 5  > 5 |  |  |  |  |
|  | 54 | 27 | 27 | 0.004^a^ |
|  | 144 | 103 | 41 |  |
| Tumor encapsulation  no  yes |  |  |  |  |
|  | 110 | 83 | 27 | 0.001^a^ |
|  | 88 | 47 | 41 |  |
| Vascular invasion  no  yes |  |  |  |  |
|  | 88 | 42 | 46 | <0.0001^a^ |
|  | 110 | 88 | 22 |  |
| Tumor differentiation (Edmondson-Steiner)  I-II  III-IV |  |  |  |  |
|  | 99 | 54 | 45 | 0.001^a^ |
|  | 99 | 76 | 23 |  |
| Tumor stage (AJCC)  I-II  III-IV |  |  |  |  |
|  | 131 | 76 | 55 | 0.002^a^ |
|  | 67 | 54 | 13 |  |

^a^ Significant difference; ^b^ a patients with HCV infection was excluded.
