# Supplementary material for: PHF8 upregulation contributes to autophagic degradation of E-cadherin, epithelial-mesenchymal transition and metastasis in hepatocellular carcinoma
Source: J Exp Clin Cancer Res. 2018 Sep 4;37:215. doi: 10.1186/s13046-018-0890-4 (PMC6122561; doi:10.1186/s13046-018-0890-4)
Supplement: Supplementary file 6 — Table S5. Univariate- and Multivariate- analysis of risk factors for relapse-free survival (RFS) and overall survival (OS). (DOCX 18 kb) [file 13046_2018_890_MOESM6_ESM.docx]

Table S5. Univariate- and Multivariate- analysis of risk factors for relapse-free survival (RFS) and overall survival (OS).

| Variable | Univariate analysis | | Multivariate analysis | |
| --- | --- | --- | --- | --- |
|  | HR (95% CI) | *P* Value | HR (95% CI) | *P* Value |
| RFS |  |  |  |  |
| PHF8 (high vs low) | 0.328 (0.213-0.505) | <0.0001^a^ | 0.451 (0.286- 0.712) | 0.001^a^ |
| Gender (male vs female) | 1.406 (0.820- 2.410) | 0.215 |  |  |
| Age, y (> 50 vs ≤ 50) | 1.354 (0.939- 1.952) | 0.105 |  |  |
| Cirrhosis (yes vs no) | 0.737 (0.457- 1.187) | 0.209 |  |  |
| HBsAg (positive vs negative) | 0.839 (0.521- 1.352) | 0.471 |  |  |
| Preoperative AFP, ng/mL (> 20 vs ≤ 20) | 0.683 (0.473- 0.987) | 0.042^a^ | 0.777 (0.529- 1.142) | 0.199 |
| Tumor number (multiple, ≥2 vs single) | 0.631 (0.435- 0.917) | 0.016^a^ | 1.032 (0.667- 1.597) | 0.888 |
| Maximal tumor size, cm (> 5 vs ≤ 5) | 0.590 (0.392- 0.890) | 0.012^a^ | 0.935 (0.594-1.470) | 0.769 |
| Tumor encapsulation (yes vs no) | 1.928 (1.346- 2.762) | 0.0003^a^ | 1.016 (0.669- 1.544) | 0.940 |
| Vascular invasion (yes vs no) | 0.343 (0.236- 0.499) | <0.0001^a^ | 0.533 (0.342- 0.832) | 0.006^a^ |
| Tumor differentiation (Edmondson-Steiner, III-IV vs I-II) | 0.601 (0.424- 0.852) | 0.004^a^ | 0.832 (0.570- 1.214) | 0.340 |
| Tumor stage (AJCC, III-IV vs I-II) | 0.375 (0.264- 0.534) | <0.0001^a^ | 0.475 (0.310- 0.728) | 0.001^a^ |
| OS |  |  |  |  |
| PHF8 (high vs low) | 0.379 (0.232- 0.620) | <0.0001^a^ | 0.545 (0.325- 0.915) | 0.022^a^ |
| Gender (male vs female) | 1.524 (0.850- 2.731) | 0.158 |  |  |
| Age, y (> 50 vs ≤ 50) | 1.388 (0.917- 2.103) | 0.122 |  |  |
| Cirrhosis (yes vs no) | 0.753 (0.434- 1.304) | 0.311 |  |  |
| HBsAg (positive vs negative) | 1.249 (0.764- 2.040) | 0.376 |  |  |
| Preoperative AFP, ng/mL (> 20 vs ≤ 20) | 0.600 (0.387- 0.930) | 0.022^a^ | 0.751 (0.476- 1.186) | 0.219 |
| Tumor number (multiple, ≥2 vs single) | 0.629 (0.411- 0.963) | 0.033^a^ | 1.177 (0.715- 1.938) | 0.522 |
| Maximal tumor size, cm (> 5 vs ≤ 5) | 0.445 (0.266- 0.745) | 0.002^a^ | 0.702 (0.400- 1.231) | 0.217 |
| Tumor encapsulation (yes vs no) | 1.786 (1.183- 2.696) | 0.006^a^ | 0.947 (0.589- 1.523) | 0.824 |
| Vascular invasion (yes vs no) | 0.375 (0.244- 0.576) | <0.0001^a^ | 0.588 (0.355- 0.977) | 0.040^a^ |
| Tumor differentiation (Edmondson-Steiner, III-IV vs I-II) | 0.532 (0.355- 0.798) | 0.002^a^ | 0.739 (0.475- 1.148) | 0.178 |
| Tumor stage (AJCC, III-IV vs I-II) | 0.324 (0.217- 0.483) | <0.0001^a^ | 0.448 (0.272- 0.740) | 0.002^a^ |

^a^ Significant difference
