# Supplementary material for: PHF8 upregulation contributes to autophagic degradation of E-cadherin, epithelial-mesenchymal transition and metastasis in hepatocellular carcinoma
Source: J Exp Clin Cancer Res. 2018 Sep 4;37:215. doi: 10.1186/s13046-018-0890-4 (PMC6122561; doi:10.1186/s13046-018-0890-4)
Supplement: Supplementary file 9 — Table S6. Correlation of PHF8 expression with expression of ATG17/ FIP200 and E-cadherin based on immunohistochemistry analysis. (DOCX 14 kb) [file 13046_2018_890_MOESM9_ESM.docx]

Table S6. Correlation of PHF8 expression with expression of ATG17/ FIP200 and E-cadherin based on immunohistochemistry analysis

|  | PHF8 expression | | *P* value |
| --- | --- | --- | --- |
|  | high | low |  |
| FIP200 expression |  |  |  |
| high | 111 | 20 |  |
| low | 19 | 48 | <0.0001^a^ |
| E-cadherin expression |  |  |  |
| high | 106 | 29 |  |
| low | 24 | 39 | <0.0001^a^ |

^a^ Significant difference
