# Supplementary figures and images for: Correction to: PHF8 upregulation contributes to autophagic degradation of E-cadherin, epithelial-mesenchymal transition and metastasis in hepatocellular carcinoma
Source: J Exp Clin Cancer Res. 2018 Nov 7;37:270. doi: 10.1186/s13046-018-0944-7 (PMC6220448; doi:10.1186/s13046-018-0944-7)

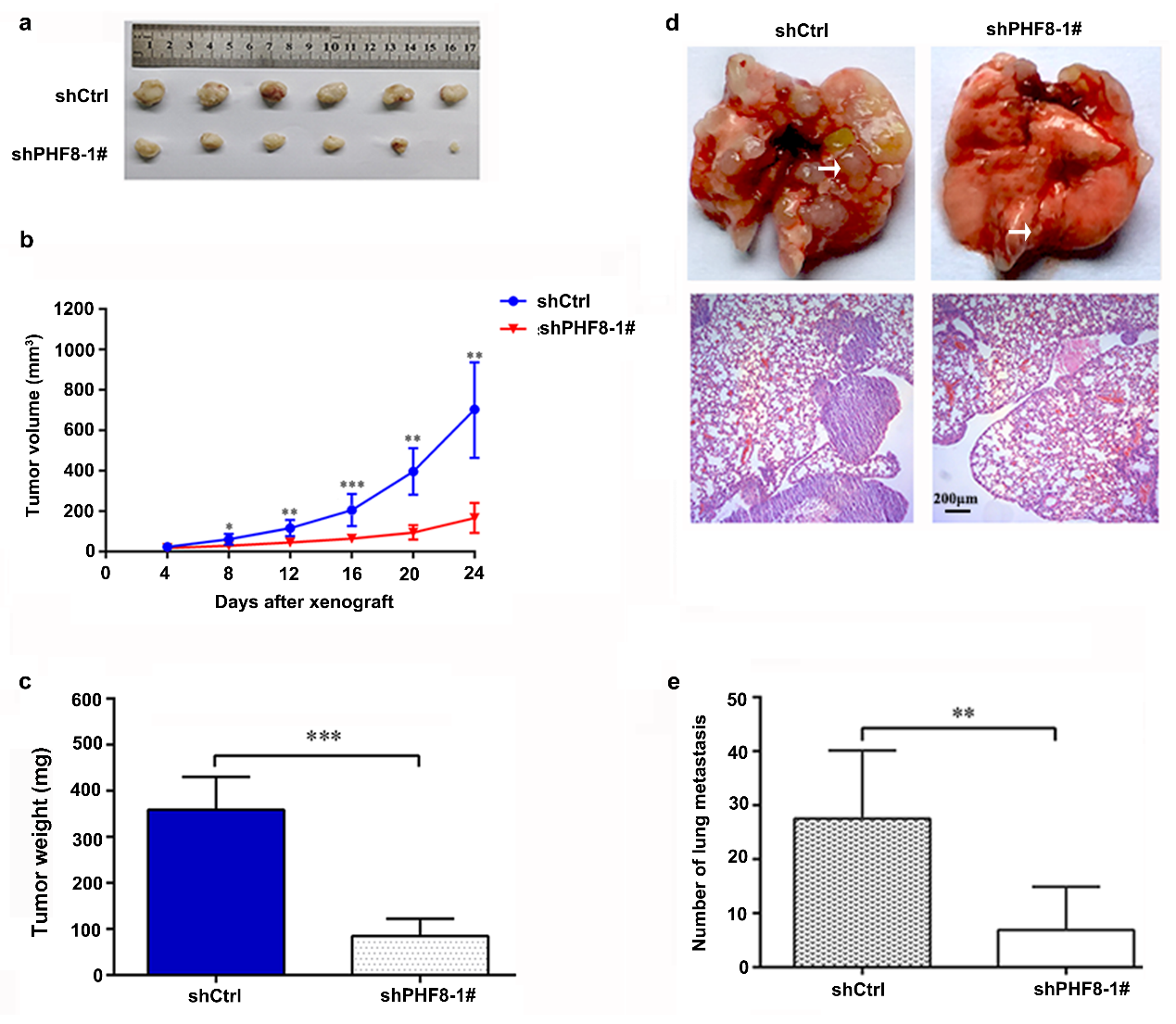

Supplement: Supplementary file 1 — Figure S3. The blockage of PHF8 inhibits tumorigenesis and metastasis in vivo. a – d) Appearance of primary tumor, tumor growth curves and tumor weight in two groups (n = 6). d Overview of lung metastatic lesions (upper panel, white arrow indicated the metastatic colonization) and HE images (lower panel, magnification, × 100). e The number of lung metastatic nets of each group was counted in a low power field (n = 6). * P < 0.05, ** P < 0.01, *** P < 0.001. Data were presented by mean ± SD. (DOCX 956 kb) [file 13046_2018_944_MOESM1_ESM.docx]
